# Emerging and future strategies in the management of recalcitrant *Candida auris*

**DOI:** 10.1093/mmy/myac008

**Published:** 2022-02-10

**Authors:** Nihal Bandara, Lakshman Samaranayake

**Affiliations:** Bristol Dental School, University of Bristol BS1 2LY, Bristol, United Kingdom; Faculty of Dentistry, the University of Hong Kong, Hong Kong SAR, China

**Keywords:** Candida auris, antimicrobial resistance, novel therapeutics, biofilms, antifungals

## Abstract

*Candida auris* is an emerging, multi drug resistant fungal pathogen that has caused infectious outbreaks in over 45 countries since its first isolation over a decade ago, leading to in-hospital crude mortality rates as high as 72%. The fungus is also acclimated to disinfection procedures and persists for weeks in nosocomial ecosystems. Alarmingly, the outbreaks of *C. auris* infections in Coronavirus Disease-2019 (COVID-19) patients have also been reported. The pathogenicity, drug resistance and global spread of *C. auris* have led to an urgent exploration of novel, candidate antifungal agents for *C. auris* therapeutics. This narrative review codifies the emerging data on the following new/emerging antifungal compounds and strategies: antimicrobial peptides, combinational therapy, immunotherapy, metals and nano particles, natural compounds, and repurposed drugs. Encouragingly, a vast majority of these exhibit excellent anti- *C. auris* properties, with promising drugs now in the pipeline in various stages of development. Nevertheless, further research on the modes of action, toxicity, and the dosage of the new formulations are warranted. Studies are needed with representation from all five *C. auris* clades, so as to produce data of grater relevance, and broader significance and validity.

**Lay Summary:**

Elimination of *Candida auris* that causes deadly infections to susceptible individuals is extremely challenging due to the lack of effective treatment options. Promising, new antifungal agents and strategies are being developed and further refinement will facilitate their clinical use in the near future.

## Introduction

With the arrival of Coronavirus Disease -2019 (COVID-19) pandemic, and the associated superinfections, such as mucormycosis, there is a renewed interest in another rapidly escalating, silent pandemic of antimicrobial resistance (AMR). The latter phenomenon has been declared by the World Health Organisation,^1^ as one of the top ten global public health threats facing humanity today. It has been estimated that there will be over 10 million deaths associated with AMR infections, and its cumulative global economic burden will increase up to ${\$}$100.2 trillion by 2050, if immediate action is not taken to counter the crisis (https://amr-review.org/).^2^ The AMR research and surveillance to date has been largely focused on antibacterial, antiviral, and antimalarial resistance. Nevertheless, it is well known that fungal infections too are a significant contributor to AMR.^1^ The recent surges in systemic fungal infections, particularly in immunocompromised populations, and those due to pan-resistant *Candida auris* as well as the current ‘black fungus’ disease associated with COVID-19 due to *Mucorales* species, are examples of fungal diseases that are resurging due to the sparsity of effective antifungals.^3^

Since its first isolation in 2009, from an ear discharge of a female patient in Japan,^4^*C. auris* infections have been reported in 45 countries in far-Eastern Asia, the Middle East, Africa, Europe, North America and South America^5^ highlighting its alarming global spread. Due to its rapid emergence within the past decade or so, the Centers for Disease Control and Prevention (CDC) European Centre for Disease Prevention and Control (ECDC), and Public Health England have released a clinical alert to healthcare facilities in 2016 to warn of the global threat of pan-resistant *C. auris*.^6–8^ Outbreaks of *C. auris* associated with COVID-19 continue to be in the limelight, as we write.^9–12^

The origins of *C. auris* have been intriguing. In-depth analyses of 18S internal transcribed spacer (ITS) and rDNA sequences of the 28S D1/D2 regions and sequences of 50 different proteins have revealed that *C. auris* is a member of the *Metschnikowiaceae* family within the *Candida/Clavispora* clade.^4,13,14^ Based on their genetic and genomic information and first isolated locations, *C. auris* has been clustered into four major discrete genetic clades (Clades I-IV): the South Asian Clade, the East Asian Clade, the South African Clade, and the South American Clade.^15^ A fifth Clade originated from Iran, separated from the other clades by over 200 000 SNPs, has been recently reported.^16^ Pitfalls in disparate identification techniques, reporting, and surveillance of *C. auris*, may have underestimated its prevalence and genomic diversity, hence, caution must be exercised when interpreting the current epidemiological data of *C. auris* infections.

The commonest human habitat of *C. auris* is the skin, however, several studies have reported their isolation from the gut, and the oral, and esophageal mucosae of infected individuals.^15^ Similar to other major *Candida* pathogens, *C. auris* primarily infects the usual spectrum of compromised individuals including those with uncontrolled diabetes mellitus, chronic renal diseases, neutropenia, and those on immunosuppressive therapy, broad-spectrum antimicrobials, and those with indwelling medical devices, or at extremes of age.^17^ Although, first isolated from an ear infection, *C. auris* is now known to cause an array of human diseases ranging from fungemias, surgical/nonsurgical wound infections, urinary tract infections, meningitis, myocarditis, skin abscesses, to bone infections.^18–21^

Biologically, the behavior of *C. auris* is similar to most other *Candida* pathogens such as *C. albicans, C. tropicalis, C. parapsilosis* of the, so called, CTG clade [Species that translate the CTG codon into serine instead of leucine^22^] possessing shared virulence attributes such as biofilm formation, yeast to hyphae transition, and phenotypic switching.^23,24^ Hence, these characteristics are likely to modulate *C. auris* virulence including antifungal tolerance, and survival in diverse habitats in the host, as well as on in inert, abiotic, environmental surfaces.

## *C. auris* antifungal resistance

The major reason why *C. auris* has been in the spotlight, is their notorious and dogged antifungal resistance, relative to other *Candida* species, and consequent persistence causing chronic infections with poor prognosis, particularly in compromised hosts. Indeed, the consensus is that *C. auris* associated candidemias and septicemias could lead to in-hospital crude mortality rates as high as 72%.^25^

A study by Lockhart *et al.* (2017) clearly illustrates the alarming breadth and depth of this problem.^15^ They noted that almost one half of all *C. auris* isolates from various studies were resistant to the most widely used, azole antifungal, fluconazole (44.29% resistance), followed by the polyene, amphotericin B (15.46%), and other antifungal drugs such as voriconazole (12.67%), caspofungin (3.48%) and flucytosine (1.95%).^26^ Thankfully, it appears that the yeast is still reasonably sensitive to echinocandin, and the DNA-analog flucytosine with only a small minority of 5% isolates being, disconcertingly pan-resistant, thus far.

*C. auris* employs several different molecular mechanisms to evade the action of antifungals. In brief, the azole resistance is associated with the overexpression of drug efflux pumps belonging to ATP Binding Cassette (ABC) and Major Facilitator Superfamily (MFS) transporters, and alterations of the ergosterol synthesis pathway (overexpression of *ERG11*, and point mutations in *ERG11*, i.e., Y132F or K143R). The echinocandin resistance in *C. auris* is shown to be attributable to mutations of *FKS1*, a gene that codes the enzyme responsible for the key fungal cell wall component, β(1,3)D-glucan. Single nucleotide polymorphisms in genes related to the ergosterol synthesis pathway leading to altered sterol composition and potential amino acids substitution in the *FUR1* gene (i.e., F211I) have been linked to *C. auris* resistance to polyenes (e.g., amphotericin B) and nucleoside analogs (e.g., flucytosine) respectively.^27–36^ Emerging evidence suggests novel mechanisms such as mutations in the zinc-cluster transcription factor-encoding gene *TAC1B* may also have a role in *C. auris* antifungal resistance.^37^ Further in-depth discussion of the resistance mechanisms of *C. auris* is beyond the remit of this review and could be found elsewhere.^38,39^ The principal mechanisms of resistance are summarized in Table 1.

**Table 1.** Antifungal resistance mechanisms of *C. auris*.

Antifungal class	Mode of action	Resistance mechanisms	References
Azole	Inhibit lanosterol 14 α-demethylase (Erg11p) in during the synthesis of ergosterol leading to formation of defective cell membrane and accumulation of toxic sterol metabolites	Point mutations in the lanosterol 14 α-demethylase (*ERG11*) gene; substitutions of *Y132F, K143R*, and *F126L* Upregulation of the *ERG11* expression Increased copy number of *ERG11* Overexpression of CDR genes members of the ATP-binding cassette (ABC) family and *MDR1* member of the major facilitator superfamily (MFS) transporters Increased copy numbers of transcription factor that regulate expression of ABC and MFS family transporters	^14,29,244^
Echinocandins	Inhibits 1,3-beta-glucan synthase leading to defective cell wall formation	Mutations in the *FKS1* gene encoding 1,3-beta-glucan synthase *FKS1* gene substitutions S639F, S639P, S639Y, S652Y	^29,35,36^
Polyene	Binds to the membrane ergosterol and forms multimeric pores in the plasma membrane	Single nucleotide polymorphisms (SNPs) in different genomic loci	^32^
Nucleoside analogs	Competitive inhibition of purine and pyrimidine uptake Incorporation into fungal RNA to inhibit DNA and RNA synthesis	Amino acid substitution F211I in the *FUR1* gene	^36^

The alarming severity of the infection, and the associated global morbidity and mortality rates, coupled with the emergence of the resistance to multiple antifungal classes, have all led to an urgent exploration of novel, and promising candidate antifungal agents for *C. auris* therapeutics. The main objective of this narrative review, therefore, is to codify the emerging data on the new and emerging antifungal classes for *C. auris.* For ease of review, we discuss these in alphabetical order in the following sections. The main findings are also summarized in Table 2.

**Table 2.** Summary of anti-*C. auris* agents, their proposed actions, and efficacies.

Therapeutic group	Anti-*C. auris* agent	Mode of action	Efficacy	Number of isolates tested	Reference
Antimicrobial combination therapy	Antifungal combinations: echinocandins with azoles (Ongoing clinical trial against non-*Candida* fungal infection)	Azoles inhibit 14α-demethylase during ergosterol synthesis to compromise cell membrane integrity and lead to accumulation of toxic sterol intermediates. Echinocandins: inhibit glucan synthesis by supressing beta-1,3-D-glucan synthase leading to a compromised cell wall and subsequent cell lysis	Synergy between micafungin and voriconazole fractional inhibitory concentration index (FICI) <0.5. MIC of micafungin single vs. combined (0.125-8 μg/ml 0.016–2 μg/ml); MIC of voriconazole single vs. combined (0.5–8 μg/ml 0.125–1 μg/ml)	10	^42^
			Co-delivery of anidulafungin with voriconazole demonstrated synergy against 5 *Candida auris* strains and partial synergy against 22 strains. Co-delivery of anidulafungin with isavuconazole demonstrated synergy against 11 *Candida auris* strains and partial synergy against 19 strains	36	^46^
	Antifungal combinations: 5-fluorocytosine with azoles	5-fluorocytosine inhibits RNA and DNA synthesis by incorporating 5-fluorouracil into fungal RNA.	Improved MIC when 5-fluorocytosine 1 μg/ml combined with voriconazole (>2 μg/ml vs. 0.015 μg/ml)	13	^44^
			In the presence of 0.91 μM flucytosine, the IC_50_ value for voriconazole decreased from 7.2 to 2.9 μM. In the presence of 0.55 μM flucytosine, IC_50_ value of posaconazole decreased from 0.45 to 0.15 μM.	1	^45^
	Antifungal combinations: 5-fluorocytosine with echinocandins		Improved MIC when 5-fluorocytosine 1 μg/ml combined with anidulafungin (4 μg/ml vs. 0.0078 μg/ml) caspofungin (2 μg/ml vs. 0.0078 μg/ml) or micafungin (4 μg/ml vs. 0.0078 μg/ml)	6	^44^
	Antifungal combinations: 5-fluorocytosine with polyenes	Polyenes: bind to ergosterol in cell membrane leading to pore formation and leakage of cellular cations and anions, and fungal cell death	Improved MIC when 5-fluorocytosine 1 μg/ml combined with amphotericin B (≥2 μg/ml vs. 0.25 μg/ml)	9	^44^
	Antifungal and antibiotic combinations: azoles with sulfonamides	Sulfamethoxazole inhibits bacterial folate synthesis leading to the inhibition of bacterial purines and DNA synthesis. The mechanism behind its synergy with azoles is not known.	Co-delivery of fluconazole with sulfamethoxazole demonstrated synergy against 1 *Candida auris* strain (fluconazole MIC 16 μg/ml vs. 4 μg/ml, sulfamethoxazole MIC 512 μg/ml vs. 16 μg/ml) Co-delivery of voriconazole with sulfamethoxazole demonstrated synergy against 3 *Candida auris* strain (voriconazole MIC 1–8 μg/ml vs. 0.06–2 μg/ml, sulfamethoxazole MIC 512 μg/ml vs. 16–128 μg/ml Co-delivery of itraconazole with sulfamethoxazole demonstrated synergy against 3 *Candida auris* strain (itraconazole MIC 1–2 μg/ml vs. 0.25–0.31 μg/ml, sulfamethoxazole MIC 512 μg/ml vs. 16-32 μg/ml)	3	^47^
	Antifungal and antibiotic combinations: azoles with colistin	Colistin affects the bacterial cytoplasmic membrane, changing its permeability and disrupting the cell membrane. The mechanism behind its synergy with azoles is not known.	Co-delivery of isavuconazole and colistin exhibited synergy against *Candida auris* (isavuconazole MIC 0.004–0.5 μg/ml vs. 0.001–0.25 μg/ml, colistin MIC 128 μg/ml vs. 8–32 μg/ml)	15	^50^
	Antifungal and non-antimicrobial drug combinations: azoles with pitavastatin	Pitavastatin competitively inhibit HMG-CoA (3-hydroxy-3-methylglutaryl coenzyme A) reductase, that catalyses the conversion of HMG-CoA to mevalonate, to inhibit cholesterol biosynthesis. The mechanism behind its synergy with azoles is not known.	Co-delivery of fluconazole and pitavastatin exhibited synergy against *Candida auris* (fluconazole MIC 256 μg/ml vs. 4–16 μg/ml, colistin MIC 64–128 μg/ml vs. 8–32 μg/ml	5	^51^
	Antifungal and non-antimicrobial drug combinations: azoles with ospemifene	Ospemifene is a selective estrogen receptor modulator that selectively binds to estrogen receptors to stimulate/inhibit the activity of estrogen in humans. Increased activity of itraconazole is likely to be associated with the increased affinity of ospemifene to multidrug efflux pumps	Co-delivery of itraconazole and ospemifene exhibited synergy against *Candida auris* (itraconazole MIC 0.5-1 μg/ml vs. 0.125–0.25 μg/ml, ospemifene MIC 256 μg/ml vs. 4 μg/ml	5	^53^
	Antifungal and non-antimicrobial drug combinations: azoles with aprepitant	Aprepitant is an antiemetic that antagonise substance P/neurokinin 1 (NK1) receptors in humans. Azoles with aprepitant combination may affect *C. auris* membrane transport processes, ions homeostasis and subsequent ROS detoxifying mechanisms and ergosterol biosynthesis, and fungal glucose transport.	Co-delivery of aprepitant with fluconazole (n = 4), itraconazole (n = 8) or voriconazole (n = 2) exhibited synergy against *Candida auris* (fluconazole MIC 1–256 μg/ml vs. 0.5–8 μg/ml, itraconazole MIC 0.125–1 μg/ml vs. 0.0312–0.125 μg/ml, voriconazole MIC 0.0078–4 μg/ml vs. 0.062 μg/ml aprepitant MIC > 128 μg/ml vs. 0.5–8 μg/ml)	10	^52^
	Antifungal and non-antimicrobial drug combinations: azoles with lopinavir	Lopinavir inhibits the activity of HIV-1 protease enzyme that is critical for the HIV viral lifecycle. Azoles with lopinavir combination may affect *C. auris* membrane transport processes, ions homeostasis and subsequent ROS detoxifying mechanisms and ergosterol biosynthesis, and fungal glucose transport.	Co-delivery of lopinavir with fluconazole (n = 3), itraconazole (n = 10) or voriconazole (n = 6) exhibited synergy against *Candida auris* (fluconazole MIC 1–256 μg/ml vs. 0.25–32 μg/ml, itraconazole MIC 0.125–1 μg/ml vs. 0.00098–0.0078 μg/ml, voriconazole MIC 0.0078–4 μg/ml vs. 0.0156–0.5 μg/ml lopinavir MIC > 128 μg/ml vs. 1–8 μg/ml)	10	^54^
	Antifungal and non-antimicrobial compounds combinations: 5-fluorocytosine with myriocin	Myriocin is a serine palmitoyltransferase inhibitor that impede sphingolipid biosynthesis in eukaryotic cells. The mechanism behind its synergy with 5-fluorocytosine is not known.	In the presence of 0.55–0.91 μM flucytosine, the IC_50_ value for Myriocin decreased from 0.63–2.2 μM to 0.18–0.62 μM.	3	^45^
	Antifungal and non-antimicrobial compounds combinations: antifungal drugs with *Neosartorya fischeri* antifungal protein 2; NFAP2	NFAP2 is a small, cysteine-rich, cationic antifungal protein that is likely to kill *Candida spp.* via membrane disruption. The mechanism behind its synergy with approved antifungals is not known.	Co-delivery of NFAP2 significantly lowered the MBICs of fluconazole (32- to 128-fold), amphotericin B (4- to 64-fold), anidulafungin (16- to 128-fold), caspofungin (4- to 128-fold), and micafungin (64- to 128-fold).	5	^56^
	Antifungal and antiseptic combinations: Azoles and domiphen bromide	Domiphen bromide is a cationic surfactant which possibly increases the efficacy of azoles by increasing the permeability of the vacuolar membrane, thereby releasing sequestered azoles.	Co-delivery of to 150 μM of miconazole and 37.5 μM domiphen bromide decreased *Candida auris* biofilm viability by ∼3 Log_10_ CFUs	1	^57^
Antimicrobial peptides	PepBiotics	Interfere with metabolic activity, fungal growth, and/or viability	Complete suppression activity at <1 μM	1	^72^
	lipopeptide-AF_4_	Structural homologue of bacillomycin D which induce ion-conducting pores in the lipid component of the fungal cell membranes and subsequent cell death.	Planktonic MIC: 3.48 μg/ml Biofilm MIC: 2-4 folds of planktonic MIC	10	^73^
	Cm-p5	Not known.	MIC 11 μg/ml (free form) MIC < 10 μg/ml in hydrogel form	1	^77^
	Crotamine	Not known. Functional similarities with human β defensins	MIC_50_ ∼ 40–160 μM	5	^80^
	θ defensins	Induce oxidative stress and accumulation of ROS within the fungus.	MICs 3.125–6.25 μg/ml	2	^96^
	Histatin 5	Likely to act on multiple intracellular targets leading to nonoxidative events such as intracellular ions leakage, ion imbalance, and volume loss accompanied by vacuolar disruption.	MIC_90_ 7.5 μM	10	^97^
	Ceragenins (CSA131)	Potentially via membrane perturbation, damage via reactive oxygen species (ROS) and attenuation of mitochondrial functions leading to apoptosis	MIC_50_ 0.5–1 μg/ml MIC_90_ 1 μg/ml MBIC_50_ 2–4 μg/ml MBIC_80_ 4–64 μg/ml	107 (planktonic) 5 (biofilm)	^99,102,103^
Immunotherapy	Anti-CR3-RP polyclonal antibody	Not known	1;100 dilution; biofilm formation (36–73% inhibition) and established biofilms (28–46% inhibition)	3	^106^
	Anti-Als3p antibody generated via vaccinating mice with NDV-3A vaccine (based on the N-terminus of Als3 protein)	Enhances macrophage-mediated killing, supresses biofilm formation	Sera from NDV-3A-vaccinated mice (1:10 dilution): 40% increase in macrophage mediated *Candida auris* killing and 40% survival of mice with *Candida auris* candidemia	5	^107^
	Human uterine cervical stem cells conditioned medium (hUCESC-CM)	Not known	Up to 56% of growth suppression	2	^108^
Metals and nanoparticles	Gallium	Gallium replaces iron in iron containing proteins to alter the functionality of the protein. This leads to arresting the cellular metabolism and growth	MICs 128–256 μg/ml	8	^114^
	Gold(I)−phosphine complexes	Not fully understood. Gold complexes may inhibit mitochondrial functions of the fungus	MIC of chiral square-planar gold(I) complexes MIC 0.98–7.8 μg/ml and MBIC_90_ 3.9 μg/ml for forming biofilms and 7.8–15.6 μg/ml against preformed biofilms	2	^118^
	Silver nanoparticles (Completed clinical trial against non-*Candida* fungal infections)	Exact mechanism is not known. They are likely to attach yeast cell surface, increase the cell wall/membrane permeability, and disrupt the cell membrane integrity, leading to cellular apoptosis. In addition, reduction of cell wall ergosterol and hydrolytic enzyme production in other *Candida spp.* have been noted	IC_50_ of 0.06 μg/ml (0.06 ppm) for biofilm formation, and 0.48 μg/ml (0.48 ppm) for preformed biofilms	1	^123^
			MIC < 0.5–1 μg/ml, MFC 1- ≤ 32 μg/ml IC_50_ of 0.5–4.9 μg/ml for biofilm formation, and 1.2–6.2 μg/ml for preformed biofilms	10	^124^
	Bismuth nanoparticles	Exact mechanism is not known. Likely to alter cell membrane permeability.	MIC 1–4 μg/ml; the IC_50_ for biofilm formation 5.1–113.1 μg/ml	10	^129^
	Silver nanoparticles with curcumin	Curcumin is shown to downregulate Δ^5,6^ desaturase (*ERG3*) leading to significantly lower ergosterol and accumulation of toxic sterol intermediates which leads to cell death. Also reduces proteinase secretion and alter ATPase activity in fungi.	Silver nanoparticles loaded with curcumin: hydroxypropyl-β-cyclodextrin showed significant reduction of *Candida auris* in disc diffusion assay	1	^134^
	Ag-Cu-Co trimetallic nanoparticles	Likely to induce cellular apoptosis and subsequent cell necrosis. Also shown to arrest fungal cell cycle	MIC range of 0.39–0.78 μg/ml	25	^131^
			10 mg/ml nanoparticles treatment reduced planktonic CFU by 1.49–10.2 log10 and biofilm CFU by 0.98–9.68 log10	6	^138^
Miscellaneous drugs/compounds	Phenylthiazole compounds	Not known	Planktonic MIC 2 μg/ml >90% reduction in biofilm formation at 2 μg/ml and >50% reduction in preformed biofilms at 8 μg/ml	8	^140^
	Oxadiazolylthiazoles	Not known	Planktonic MIC 2–4 μg/ml	3	^141^
	MYC-053	Inhibits chitin synthesis by blocking chitin synthase, leading to defective fungal cell wall and inhibits nucleic acid synthesis in fungi.	IC_50_ 1–4 μg/ml MIC 4 μg/ml	5	^142^
	VT-1598 (Completed clinical trial against non-*Candida* fungal infections)	Inhibits the production of ergosterol by acting on the fungal Cyp51 enzyme.	MIC range 0.03–8 μg/ml (MIC_50_ 0.25 μg/ml and MIC_90_ 1 μg/ml) When treated with up to 50 mg/kg, a longer survival rates (>21 days) and lower fungal burdens in the kidneys of neutropenic murine model infected with *Candida auris* (mean log_10_ CFU/g, treated vs. control: 3.67 vs7.26)	100	^146^
	Arylamidine T-2307	Trigger mitochondrial membrane collapse in fungi.	MIC50 0.008 to 0.015 μg/ml, and 100% inhibition at 0.25 to >4 μg/ml. Significant reductions in kidney CFU in mice treated at 3 mg/kg (mean 5.06 log_10_ CFU/g)	23	^149^
	Drimenol	Likely to affect fungal protein secretion, vacuolar functions, chromatin remodelling and cyclin dependent protein kinase (CDK)-associated functions.	MIC 30 μg/ml; complete inhibition MIC 50 μg/ml	1	^151^
	Cuminaldehyde derivative	Not known.	MIC_50_ 2–15 μg/ml	1	^154^
	Amidinourease compounds	Not fully understood; may involve in its uptake and intracellular accumulation within the fungus.	MIC 8–64 μg/ml MBIC 128–256 μg/ml	18	^155^
	Aryl- and heteroaryl-substituted hydrazones	Not fully understood. Likely to interfere with fungal DNA-protein interactions.	MIC 0.015–7.8 μg/ml; significant suppression of biofilm formation at 15.6–31.3 μg/ml	10	^156^
	Acetohydroxyacid synthase inhibitors	Blocks the acetohydroxyacid synthase leading to the inhibition of branched-chain amino acid biosynthesis pathway.	MIC_50_ of bensulfuron methyl 0.09 μM MBIC_50_ of bensulfuron methyl and chlorimuron ethyl 0.596–1.98 μM	2	^158^
Natural compounds	Quorum sensing molecules: farnesol	Farnesol is actively involved in ergosterol biosynthesis, induce intracellular ROS, and disrupt mitochondrial functions in several *Candida* species. The mechanism of anti-*C. auris* activity is not yet known. May be associated with reduced activity of drug efflux pumps and downregulation of the genes coding for them	Significant reduction of growth rate for up to 12 h when exposed to 50–300 μM. Co-delivery of farnesol with fluconazole (fluconazole MIC_50_ > 512 μg/ml vs. 64 μg/ml, Farnesol MIC_50_ 300 μM vs. 75 μM), itraconazole (itraconazole MIC_50_ 8–32 μg/ml vs. 0.5 μg/ml, Farnesol MIC_50_ 300 μM vs. 4.69–9.38 μM), voriconazole (voriconazole MIC_50_ 64 μg/ml vs. 0.5 μg/ml, Farnesol MIC_50_ 150–300 μM vs. 4.69–9.38 μM), posaconazole (posaconazole MIC_50_ 16 μg/ml vs. 0.25 μg/ml, Farnesol MIC_50_ 150 μM vs. 2.34 μM) or isavuconazole (isavuconazole MIC_50_ 4–8 μg/ml vs. 0.125 μg/ml, Farnesol MIC_50_ 300 μM vs. 9.38–18.75 μM) exhibited synergy against *Candida auris* biofilms	3	^167^
			Co-delivery of farnesol with anidulafungin (anidulafungin MIC_50_ > 64 μg/ml vs. 1 μg/ml, Farnesol MIC_50_ 300 μM vs. 75–150 μM), caspofungin (caspofungin MIC_50_ 8→64 μg/ml vs. 1 μg/ml, Farnesol MIC_50_ 300 μM vs. 9.38–75 μM), or micafungin (micafungin MIC_50_ > 64 μg/ml vs. 1 μg/ml, Farnesol MIC_50_ 150–300 μM vs. 37.5–75 μM) exhibited synergy against *Candida auris* biofilms	4	^168^
			MIC of farnesol 62.5–125 mM. Farnesol concentrations of 125 mM inhibited *Candida auris* adhesion, 7.81 mM inhibited >50% of forming biofilms, and 500 mM inhibited 12 h and 24 h biofilms	25	^169^
	Chitosan	Not known; may be associated with direct interactions of chitosan with cell surface leading to cell death	Fungicidal concentration for planktonic cells 5–20 μg/ml; biofilm MIC_50_ 10–80 μg/ml and MIC_80_ 40–160 μg/ml	4	^23^
			Planktonic MIC 5–20 μg/ml biofilm MIC_50_ 10–80 μg/ml and MIC_80_ 40–160 μg/ml. 200 mg of chitosan/kg of body weight increased the survival rate of *Galleria mellonella* wax warm infected with *Candida auris* up to 84%	8	^172^
	Plant products: Herbal monomers	Not known; likely to be associated with either the cell wall development mechanics and/or the fungal stress response	Planktonic MICs of 64 μg/ml for sodium houttuyfonate, and 50 μg/ml for cinnamaldehyde, 256 μg/ml for berberine, jatrorrhizine, and palmatine	1	^179^
	Plant products: *trans*-cinnamaldehyde	Likely to compromise cell membrane and wall integrity	MIC and MFC 0.03% (v/v)	1	^182^
	Plant products: α-Cyperone	Not known	Growth inhibition at 150 μg/ml	1	^184^
	Plant products: 6-Shogaol	Not fully understood; likely to act on drug efflux machinery of the fungus	Planktonic MIC_50_ 16–32 μg/ml and MIC_80_ 32–64 μg/ml. >97% of inhibition of forming and preformed biofilms at 64 μg/ml	5	^185^
	Bee honey	Specific mechanism is not known; antimicrobial activity of honey is associated with its osmotic activity, low pH, the formation of H_2_O_2_, and the presence of various phytochemicals.	40% honey exposure for 24 h reduced *Candida auris* growth by 2 Log_10_	32	^189^
	Probiotics (Several completed clinical trials against non-auris *Candida* infections)	Not known; likely to be associated with secondary metabolite(s) produced by the probiotic yeasts that interfere the pathogen's life cycle; secreted probiotic short-chain fatty acids or bacteriocins or competitive inhibition of the pathogen during attachment.	Significant inhibition of *Candida auris* (up to 6 log_10_ CFU) when co-cultured with *Lactobacillus paracasei* 28.4 or exposed to crude extracts of the lactobacilli supernatant (>15 mg/ml) and its first fraction (3.75– >7.5 mg/ml)	10	^192^
			Co-inoculation of *Candida auris* strains with *Saccharomyces cerevisiae* and *Issatchenkia occidentali* resulted a 44–62% reduction in *C. auris* adhesion	5	^193^
Novel antifungal compounds	Ibrexafungerp (SCY-078) (Phase 3 clinical trial; ClinicalTrials.gov Identifier: NCT03363841)	A triterpene glucan synthase inhibitor that inhibits the synthesis of β-1,3-glucan synthase leading to defective cell wall.	MIC 0.0625–2 μg/ml (mode MIC_50_ 0.5 μg/ml and MIC_90_ 1 μg/ml)	100	^196^
			MIC 0.06–8 μg/ml (mode MIC_50_ 0.5 μg/ml)	200	^197^
			MIC_90_ 1 μg/ml; significant reduction of the viability and thickness of biofilms when exposed to 4 μg/ml of ibrexafungerp	16	^198^
			modal MIC and MIC_50_ of 0.5 μg/ml (a range of 0.06–2 μg/ml)	122	^200^
	SCY-247	Analog of SCY-078 that inhibits the synthesis of β-1,3-glucan synthase leading to defective cell wall	MIC range 0.06–1 μg/ml (MIC_50_ and MIC_90_ 0.5 μg/ml). MFC range 0.5–8 μg/ml (MFC_50_ and MFC_90_ of 4 μg/ml)	44	^204^
	Fosmanogepix (APX001/APX001A) (Phase 2 clinical trial; ClinicalTrials.gov Identifier: NCT04148287)	Targets a highly conserved fungal enzyme Gwt1 that catalyses the inositol acylation step of glycosylphosphatidylinositol (GPI) anchored cell wall mannoproteins synthesis. This in turn affects maturation and localization of fungal cell wall mannoproteins, leading to compromised cell wall integrity, defective filamentation and biofilm formation, and severe defects in fungal growth.	MIC50 0.004 μg/ml and MIC90 0.031 μg/ml the exposure of APX001 significantly increased the 16-day survival rate of *Candida auris* infected immunocompromised mice.	16	^208^
			MIC_50_ range < 0.005–0.015 μg/ml (overall modal MIC 0.005 μg/ml, MIC_50_ 0.002 μg/ml and MIC_90_ 0.008 μg/ml)	100	^209^
			MIC_50_ range 0.001–0.125 μg/ml (MIC_50_ 0.016 μg/ml and MIC_90_ 0.03 μg/ml)	122	^210^
	Rezafungin (CD101) (Currently on clinical trials against invasive candidiasis; Causative organism unspecified.	Similar to echinocandins	MIC range 0.03–8 μg/ml (mode MIC_50_ 0.125 μg/ml, MIC_90_ 0.5 μg/ml)	100	^218^
			MIC range 0.06–16 μg/ml (MIC_50_ 0.25 μg/ml, MIC_90_ 1 μg/ml)	122	^220^
			Significant reduction of *Candida auris* in kidney tissues of mice with disseminated *Candida auris* candidiasis when treated with rezafungin 20 mg/kg intraperitoneally at Day 0, 3 and 6. intravenously administration of rezafungin 400 mg/once a week would likely to meet or exceed the pharmacodynamics target for >90% of *C. auris* isolates	4	^222,223^
	PC945 (Currently on clinical trials against *Candida* lung infections; Causative organism unspecified.)	Acts on ergosterol synthesis pathway by inhibiting lanosterol 14a-demethylase enzyme coded by *ERG11.*	MIC_50_ 0.063 μg/ml and MIC_90_ 0.25 μg/ml	72	^224^
	Ebselen	Not fully understood. It is considered an antioxidant that mimic glutathione peroxidase activity and catalyse the reduction of ROS, leading to the attenuation of damage caused by oxidants and radicals.	Planktonic IC_50_ 0.2345–1.47 μg/ml, complete inhibition at 2.5 μM Biofilm IC_50_ 5.864–9.781 μg/ml	10	^225^
	Suloctidil	Not fully understood. It may act as an inhibitor of thromboxane synthase or as a thromboxane receptor antagonist.	16 μg/ml inhibited *Candida auris* growth by >78% (MIC_50_ 4–8 μg/ml, MIC_90_ 4–16 μg/ml)	7	^230^
	miltefosine	Not known. Miltefosine is an alkylphosphocholine drug originally developed as an anti-cancer drug. It may inhibit cytochrome-c oxidase within mitochondria leading to mitochondrial dysfunction and apoptosis-like cell death.	Complete elimination of planktonic growth and biofilms formation at 4 μg/ml. a 90% reduction of viability of preformed biofilms at 16 μg/ml. IC_50_ for Planktonic phase 0.9237–2.472 μg/ml, biofilm formation 1.158–6.049 μg/ml, preformed biofilms 9.144–20.98 μg/ml	10	^231^
	Iodoquinol	Not known	Complete elimination of planktonic growth at 4 μg/ml IC_50_ for Planktonic phase 0.2972–2.006 μg/ml, biofilm formation 9.159–56.02 μg/ml, preformed biofilms 38.58- >64 μg/ml	10	^231^
	Niclosamide and halogenated salicylanilide	An Anthelmintic drug. They are likely to interfere morphological transition and mitochondrial protein import machinery.	Both compounds inhibited *Candida auris* biofilms at 1 μM	1	^233^
Repurposed drugs	Disulfiram	Disulfiram blocks the oxidation of alcohol by irreversibly inactivation aldehyde dehydrogenase in human cells. This results in an accumulation of acetaldehyde in the blood causing highly unpleasant symptoms. Mechanism of antifungal effect is not known.	MIC_50_ 1 μg/ml, MIC_80_ 4–8 μg/ml MBIC_80_ 64–128 μg/ml	2	^234^
	Sertraline (Currently on clinical trials against non-*Candida* infections)	Sertraline is likely to elicit its effect of *C. auris* by binding to the Erg11p in the ergosterol biosynthesis pathway.	MIC 20–40 μg/ml; a 71% inhibition of biofilm formation at 20 μg/ml	3	^235^
	Alexidine dihydrochloride	Targets PTPMT, a mitochondrial tyrosine phosphatase in mammalian cells to drive mitochondrial apoptosis. Mechanism of antifungal effect is not known.	MIC_50_ 0.73–1.5 μg/ml, MIC_80_ 1.5 μg/ml Biofilm formation and mature biofilm inhibition concentrations: MBIC_50_ and MBIC_80_ 3–6 μg/ml	2	^238^
	Mefloquine derivatives	Antifungal activity is likely to be due to the disruption of the mitochondrial membrane, interference with mitochondrial DNA stability and disruption vacuoles.	Planktonic MIC 2–8 μg/ml Planktonic MIC against fluconazole resistant isolates 4–16 μg/ml	5	^242^

**FICI**: Fractional inhibitory concentration index, **MIC**: Minimum inhibitory concentration, **IC_50_**: 50% of maximum inhibitory concentration, **ROS**: Reactive oxygen species, **CFU**: Colony forming units, **ATP**: Adenosine triphosphate, **MBIC_50_**: The Minimal Biofilm Inhibition Concentration 50%, **MBIC_80_**: The Minimal Biofilm Inhibition Concentration 80%, **MBIC_90_**: The Minimal Biofilm Inhibition Concentration 90%, **UV-C**: Ultraviolet light -C.

## Antimicrobial combination therapy

As opposed to prokaryotic bacteria, human beings and yeasts possess structurally similar nuclear material and cell membranes and are classified as eukaryotes. Hence antifungal drugs, in general, adversely affect the host cells, due to the molecular mimicry of the drug targets of the fungi and the host cells.^39^ There is, therefore, a dearth of antifungals in comparison to a plethora of antibacterial drugs currently available to manage infectious diseases due to prokaryotes.

It is now well known that *C. auris* displays a remarkable resistance to most of the current antifungal drug classes. Although there are some variations among different clades, *C. auris* is generally resistant to azole agents, but remain susceptible to echinocandins. Hence, recommendations for safe antifungal therapy dictates, reverting back to azoles from echinocandins whenever possible, so as to prevent the development of pan resistant organisms.^40^ This is due to the higher margin of safety, and the lower adverse effects of the azole class drugs such as fluconazole.

One such well-established strategy to synergise the activity of antimicrobial drugs is combination therapy, a technique effective against difficult to treat infections such as tuberculosis.^41^ A similar approach has been evaluated in order to improve the efficacy of commonly used, first line, antifungal agents against *C. auris*. In the following section, we review these approaches, their advantages, and disadvantages as well as their potential utility against *C. auris* infections.

### Antifungal combinations

Early *in vitro* studies on combined antifungal therapy were focused on azole/echinocandin combinations against *C. auris.* Thus, Fakhim *et al.* (2017) reported promising synergy between micafungin and voriconazole against ten different (Indian) isolates of fluconazole resistant *C. auris* (3 isolates were micafungin resistant).^42^ However, no synergy or additive effect was noted between caspofungin versus fluconazole, or voriconazole, or micafungin vs. fluconazole.^42^ Similarly, micafungin and voriconazole failed to exhibit significant synergism with the nucleoside analogs 5-fluorocytosine. In another study the combination of other antifungal classes such as polyenes (amphotericin B) and nucleoside analogs (5-fluorocytosine) was not synergistic against 14/15 different isolates of *C. auris* (10 Indian, 2 South Korean, 1 Japanese).^43^

A study of 15 *C. auris* isolates from a New York outbreak has shown that such variable outcomes against antifungal combinations are due to clade and isolate specificity.^44^ For instance, amphotericin B and 5-fluorocytosine (1 μg/ml) combination elicited 100% inhibition of 9 out of 15 amphotericin B resistant *C. auris* isolate (minimum inhibitory concentration; MIC ≥ 2 μg/ml vs. 0.25 μg/ml). Also, identical doses of 5-fluorocytosine overcame echinocandin resistance of six *C. auris* isolates in combination with either anidulafungin (4 μg/ml vs. 0.0078 μg/ml), caspofungin (2 μg/ml vs. 0.0078 μg/ml) or micafungin (4 μg/ml vs. 0.0078 μg/ml). Interestingly, 5-fluorocytosine complement also improved the MIC of voriconazole by >130-fold against 13 *C. auris* isolates (>2 μg/ml vs. 0.015 μg/ml).^44^ On the contrary, and in line with some previous observations,^43,44^ other two- antifungal drug combinations tested against these isolates did not elicit a significant synergistic effect. The tested drugs included polyene (amphotericin B), echinocandins (anidulafungin, caspofungin, micafungin), azoles (voriconazole, isavuconazole, posaconazole, and itraconazole) and nucleoside analog (5-flurocytosine).

In contrast, synergy of anidulafungin with isavuconazole or voriconazole, flucytosine with voriconazole or posaconazole has been witnessed in recent research, implying the isolate specific diversity of antifungal synergism in *C. auris*.^45,46^

### Antifungal and antibiotic combinations

Eldesouky *et al.* (2018) reported increased sensitivity of azoles when paired with sulfonamides. Particularly, 37% of voriconazole resistant *C. auris* (3/8 isolates) and 75% of itraconazole resistant isolates (3/4 isolates) become sensitive to the corresponding azole when delivered with the sulfamethoxazole, *in vitro*, although co-delivery of sulfamethoxazole with fluconazole reversed the resistance of only a single fluconazole resistant isolate (1/8 isolates). The 70% survival of *Caenorhabditis elegans* infected with voriconazole resistant *C. auris* further confirmed the favorable *in vivo* activity of sulfamethoxazole–voriconazole combination.^47^

Although the exact mechanism of the synergism of the antibacterial-antifungal combination is yet to be determined, the data suggest that such inhibitory activity is likely to be due to the interruption of *C. auris* folate pathway. The sulfamethoxazole–voriconazole combination, for instance, was only effective against azole resistance associated with, either overproduction of or decreased affinity for azole target (Erg11p), but not with the overexpression of multidrug efflux pumps,^47,48^ Further, the addition of trimethoprim, (usually co-administered with sulfamethoxazole) to the sulfamethoxazole and fluconazole combinations showed synergies (27%, 3/11 isolates),^49^ whilst other antifungal and antibiotic combinations such as isavuconazole and colistin have also shown promising synergy against *C. auris*.^50^

### Antifungal and non-antimicrobial drug combinations

Arguably, the most notable approach that has been experimented to improve the efficacy of azole agents against *C. auris* is to combine them with non-antimicrobial drugs or compounds. For example, Eldesouky *et al*. (2020) reported potential synergism of pitavastatin, an antihyperlipidemic statin, with azole antifungals, against *C. auris*. The former combined with fluconazole, voriconazole or itraconazole was shown to lower the MIC by 4-16 folds in five different *C. auris* isolates (fractional inhibitory concentration indices: FICI < 0.5). However, the MIC reduction was insufficient to restore the azole sensitivity of the most *C. auris* isolates. Additionally, when tested *in vivo* on a *C. elegans* model, the pitavastatin-fluconazole drug combination lowered fluconazole resistant *C. auris* burden up to 92%. Yet, *in vitro*, the combination was only effective in suppressing biofilm development of a single *C. auris* isolate by only 41% and failed to eliminate established biofilms.^51^

In a series of further follow-up studies, Eldesouky *et al.* (2020) reported that the combined therapy of azole agents, itraconazole in particular, with various other drugs potentiate the activity of the antifungal against *C. auris*. Itraconazole combined with ospemifene, a selective oestrogen receptor modulator, usually indicated for dyspareunia, or with aprepitant, an antiemetic agent, or with lopinavir, an HIV protease inhibitor, was demonstrated to lower MIC of itraconazole sensitive and resistant *C. auris* isolates by 4 to 8-folds (n = 5, FICI 0.14–0.27), 8-folds (n = 10, FICI 0.14–0.31) and 32 to 256-fold, (n = 10, FICI 0.04–0.09) respectively. All three combinations were highly effective in lowering itraconazole resistant *C. auris* fungal load in *C. elegans* model by 96%, ∼92%, ∼88.5% respectively and extended the nematode survival significantly.^52–54^ Investigators claim that the increased activity of itraconazole is likely to be associated with the increased affinity of ospemifene to multidrug efflux pumps^53^ whereas, aprepitant/itraconazole and lopinavir/itraconazole exposure appeared to impact on *C. auris* membrane transport processes, ions homeostasis and subsequent reactive oxygen species (ROS) detoxifying mechanisms and ergosterol biosynthesis, as well as fungal glucose transport.^52,54^

Latest research on combinatorial therapies have revealed the ability of azoffluxin, a novel oxindole efflux pump inhibitor, to produce a synergistic antifungal effect by reducing *C. auris* infection in mice by lowering the fungal burden by ∼1000 – folds.^55^ This is thought to be due to enhanced intracellular fluconazole accumulation through inhibition of *CDR1*, a major multidrug efflux transporter in *Candida*.

There are several other reports showing the potential of combinatorial antifungal/non-antimicrobial drugs/compound therapy. For instance, a recent report from Cheng *et al.* (2021) has revealed a synergistic anti- *C. auris* activity of flucytosine and myriocin, a serine palmitoyltransferase inhibitor that impede sphingolipid biosynthesis in eukaryotic cells.^45^ However, myriocin combination with flucytosine, another antifungal with known toxic effects at higher doses, will need to be closely watched prior to their clinical use. This is because, a derivative of myriocin, fingolimod, approved for management for multiple sclerosis, has a significant toxicity against mammalian cells.

In contrast, combinatorial therapy of a cysteine-rich cationic protein extracted from a filamentous ascomycete, *Neosartorya fischeri* (*Neosartorya fischeri* antifungal protein 2; NFAP2) with antifungal drugs significantly lowered the minimum biofilm inhibitory concentrations (MBICs) of fluconazole (32- to 128-fold), amphotericin B (4- to 64-fold), anidulafungin (16- to 128-fold), caspofungin (4- to 128-fold), and micafungin (64- to 128-fold). The protein itself elicited only a modest to weak inhibition on *C. auris* planktonic (MIC 32–512 μg/ml) and biofilm (>512 μg/ml) phenotypes.^56^ Therefore, it appears that NFAP2 would be a promising adjunct antifungal, once its safety and efficacy, in particular for catheter lock therapies are known.

### Antifungal and antiseptic combinations

The data on antifungal and antiseptic combinatorial therapy in managing *C. auris* is scarce. A topical antifungal miconazole in combination with a well-known antiseptic quaternary ammonium compound domiphen bromide elicited significant inhibition of *C. auris* biofilms.^57^ Co-delivery of 150 μM of miconazole with 37.5 μM domiphen bromide decreased *C. auris* biofilm viability by ∼3 Log_10_ colony forming units (CFUs). The authors suggested that the surfactant properties of domiphen bromide is likely to increases the efficacy of azoles by increasing the permeability of the yeast vacuolar membrane, thereby releasing sequestered azoles. The latter combination holds much promise as a topical antifungal after relevant optimisation.

### Antifungal and miscellaneous drug combinations

Wu *et al.* (2020) demonstrated that the antiparasitic drug miltefosine, an alkyl-phospholipid analog for leishmaniasis, is fungicidal against 12 isolates of fluconazole or voriconazole resistant *C. auris* at clinically safe concentrations of 2 μg/ml.^58^ This is in contrast to its high dosage of 17.2 to 42.4 μg/ml required to manage leishmaniasis.^59,60^ Other researchers have also noted comparable MICs of miltefosine against planktonic (1-4 μg/ml) and biofilm (0.25–4 μg/ml), modes of *C. auris*, although higher concentrations were required to inhibit preformed biofilms (16–32 μg/ml) and reducing the fungal burden in *Galleria mellonella*.^61^ Nevertheless, miltefosine showed only marginal synergy with amphotericin B (FICI = 0.5) against 3/12 *C. auris* isolates *in vitro*.^58^

In a similar study, Shaban *et al*. (2020) evaluated a monoterpenoid phenolic compound carvacrol, against *C. auris* and demonstrated its antifungal attributes, as well as suppression of yeast adherence, and proteinase synthesis. Also, a combination of carvacrol and fluconazole (16% synergy), and amphotericin B and nystatin (28% synergy each) elicited a reasonable degree of synergy.^62^

There are several other reports where combinatorial antifungal drug therapies against *C. auris* have been attempted but with rather modest outcomes. These include attempts to explore synergy between antifungal drugs and antitumor agents (e.g., geldanamycin), and nonsteroidal anti-inflammatory drugs (e.g., ibuprofen, diclofenac sodium, aspirin).^63–65^

To conclude, the interesting outcomes of the elegant series of studies of Eldesouky *et al.*, and various others, show the promise of combinatorial azole/antibiotic/drug therapy in overcoming *C. auris* infections, particularly in the absence of novel, efficacious antifungal agents in the horizon. The current body of data is largely preliminary, focused on a relatively few isolates and based mainly on *in vitro* assessments. Further *in vivo* studies, incorporating a broader geographical coverage of *C. auris* isolates/clades, and the molecular basis underpinning their activity, are essential to take this field forwards.

## Antimicrobial peptides

Antimicrobial peptides (AMPs) are naturally existing, positively charged, amphipathic molecules secreted by all eukaryotes and are an integral component of the innate immune system of vertebrates.^66,67^ They have been shown to possess broad, rather nonspecific antimicrobial properties against various pathogens, and they primarily kill microbes by disrupting the microbial cellular membranes. AMPs can also target some important cellular processes such as cell wall synthesis, DNA and protein synthesis, protein folding, and multiple enzymatic activities.^68,69^

AMPs vary among different species by virtue of their amino acid sequence, size, and structure, making them an attractive option in for managing microbial infections.^67,70,71^ Up to now, AMP therapy has been largely experimented against bacterial, and not fungal, pathogens although data on fungal pathogens are slowly emerging, as discussed below.

### PepBiotics

Recently, van Eijk *et al.* (2020) demonstrated strong anti-*C. auris* activity of ‘PepBiotics’, a group of custom synthesized cathelicidin-inspired AMPs. In particular, two of these peptides, CR172 (amino acid sequence: RRWVQRWIRRWRPKVAAARRWVQRWIRRWRPKV-NH2) and CR184 (amino acid sequence: APKAMRRWVQRWIRRWRPKVFQVTGSSA-NH2) significantly suppressed *C. auris* metabolic activity, at a concentration <1 μM, suggesting their potential as an anti- *C. auris* agent.^72^

### Lipopeptide -AF_4_

Some bacteria are known to produce native peptide molecules with a broad antimicrobial spectrum.^73,74^ Of these, lipopeptides such as bacillomycin, mojavensin, mycosubtilin, iturin A, bacillopeptin, and fengycin homologues secreted by *Bacillus* species, are notable as they generate ion-conducting pores in the lipid component of the fungal cell membranes leading to cell death. Despite this attractive antimicrobial property, native lipopeptides are deemed undesirable for clinical use due to their strong hemolytic activities, and the high MICs required for microbial kill.^74^ However, Ramachandran *et al.* (2018) purified three lipopeptides (AF_3_, AF_4_, and AF_5_) from *Bacillus subtilis* RLID 12.1 with promising antifungal properties against *Candida* species, including *C. auris*, with relatively low MICs and as well as hemolytic activity.^73^ Structurally, they are homologs of bacillomycin D with a shared amino acid sequence of Asn-Pro-Tyr-Asn-Gln-Thr-Ser-Xaa, where Xaa is a β-amino fatty acid variant.^73^ The lipopeptide molecule-AF_4_, in particular, is noteworthy as it demonstrated a geometric MIC of 3.48 μg/ml against clinical *C. auris* isolates with negligible hemolysis. These three molecules also showed promising inhibitory effects against *C. auris* biofilms.

### Cm-p5

AMPs derived from primitive life forms such as sea mollusks are now known to have antifungal activity very low toxicity. One such, Cm-p5, derived from a peptide isolated from the mollusk *Cenchritis muricatus* efficiently kills *C. auris, C. albicans, C. parapsilosis, Cryptococcus neoformans*, and *Trichophyton rubrum* and demonstrated only negligible toxicity against mammalian cells.^75,76^

In order to demonstrate the antifungal effect of Cm-p5, Kubiczek *et al.* (2020) developed an elegant two-layered hydrogel containing a base layer loaded with mannose-specific lectin B (LecB) that immobilizes *C. auris* cells, and a second, sandwich layer loaded with Cm-p5. The released of free Cm-p5 in this model, was anti-candidal at an MIC of 11 μg/ml, while the loaded hydrogel was effective in eradicating *C. auris* at sub-MIC concentrations of Cm-p5.^77^ These findings suggest the hydrogel functionalized with AMPs such as Cm-p5 would likely to be an attractive solution for decontamination of chronic wound infections due to *C. auris.*

### Crotamine

New research has also shown the surprising potential of antimicrobial potency of various components of snake venom. For instance, Crotamine, a native polypeptide isolated from the venom of the South American rattlesnake *Crotalus durissus terrificus* has been studied for its antifungal properties.^78,79^ Using five different *C. auris* isolates (including four amphotericin B and a single fluconazole resistant isolates), Dal Mas *et al.* (2019) demonstrated approximately 50% of growth inhibition of all of the above strains at a crotamine concentrations of 160 μM (0.8 mg/ml), with MIC ranging from 40 to 160 μM (0.2–0.8 mg/ml).^80^ However, the weak antifungal effect of crotamine was evident by its high MIC values compared with micafungin (MIC of 0.06–0.12 μg/ml). The exact antimicrobial mechanism of Crotamine is not known, as of yet.^81,82^

Interestingly, other research has shown structural and functional similarities of crotamine to human AMP, β defensins.^83^ Particularly, crotamine and β defensins share similar avidity to lipids such as phosphatidylserine of the fungal plasma membrane, involved in cellular transport and if interfered with may lead to apoptotic cell death.^84–86^ However, further data on compatibility of crotamine with mammalian cells, their stability, and PK/PD values and adverse effects in immunocompromised individuals are essential prior to its clinical use as an anti- *C. auris* agent.

### θ defensins

The discovery of mammalian AMPs such as cathelicidins, salivary histatins and α-, β- and θ- defensins, have prompted investigators to explore their use as therapeutic agents against infections including those caused by fungi. However, the lack of data on their *in vivo* potency, safety, and stability, to date has hindered such approval.^87–91^

Interestingly, θ-defensins, an 18-amino-acid macrocyclic peptide only expressed in Old World monkeys, appear to possess remarkable antifungal activities against *C. albicans* and *Cryptococcus neoformans*.^92–95^ Basso *et al.* (2018) reported that θ-defensins isoforms from rhesus macaque (RTD-1 and RTD-2) and olive baboon (BTD-2 and BTD-8) displayed significant fungicidal properties against fluconazole and caspofungin resistant *C. auris* (MICs 3.125–6.25 μg/ml). The investigators claimed that the θ-defensins were more stable than the salivary histatin-5 (Hst 5), and up to 1 000 times more effective in antifungal assays.

θ-defensins isoforms appear to induce oxidative stress and death in *C. auris*, and yet, they are nontoxic to mice, and non-hemolytic in whole blood suggesting the possibility of their use as an anti- *C. auris* agent.^96^ Hence, antifungal properties of naturally occurring θ-defensins, with further optimizations, may prove valuable in managing multidrug resistant fungal pathogens such as *C. auris*, in future.

### Histatin 5

Although less stable than θ-defensins, histatin-5 (Hst 5) is a well-recognized salivary cationic peptide with anti-candidal properties. Its effect against *C. auris* infections has been investigated by Pathirana *et al.* (2018) who observed up to 90% killing of 7 out of 10 *C. auris* isolates tested, on exposure to 7.5 μM of Hst 5 for a period of 1 h. Hst 5 appeared to be rapidly translocated into the yeast cells which led to their rapid kill, as seen with *C. albicans*.^97^ Nevertheless, due to the higher threshold of oxidative stress tolerance of *C. auris*, it is unlikely to respond as well as *C. albicans in vivo.* Similarly, the systemic stability of Hst 5 may be diminished by blood serum components and salts, and therefore it may be less likely to be effective in managing systemic *C. auris* infections such as in sepsis.^97^ However, considering its natural presence in saliva, the antifungal properties of Hst 5 may be valuable for topical management of mucosal *C. auris* infections, perhaps particularly as an antiseptic against oral candidiasis.

### Ceragenins

Despite their potent antifungal properties, a major impediment for the clinical use of AMPs has been the relatively high cost of their large-scale synthesis/preparation. To overcome this limitation, some researchers have investigated the possibility of developing low cost, non-peptide molecules to mimic the amphiphilic morphology of common AMPs.^98,99^ Molecules of this class, ceragenins, have been shown to mimic ‘secondary’ activities of AMPs during wound healing.^100^

Ceragenins possess broad-spectrum antimicrobial properties and have been tested against *C. auris*.^101,102^ The MIC of ceragenins- CSA-131 ranged from 0.5 to 1 μg/ml against 107 different isolates of *C. auris* regardless of their clade and or the antifungal susceptibility. Two tested ceragenins (CSA-44 and CSA-131) demonstrated potent antibiofilm activity against all *C. auris* isolates studied (n = 5; MBIC50 = 2-4 μg/ml, MBIC80 = 4-64 μg/ml) and a significant reduction of *C. auris* CFUs (by ∼3-fold log_10_) in porcine vaginal mucosal explants infected with *C. auris*.^103^

As the favorable tolerability of ceragenins have been established,^104^ further investigations into its molecular mechanisms of actions and *in vivo* pharmacokinetics and pharmacodynamics will likely assist their application as a future, cost-effective anti- *C. auris* agent, particularly against variants that are nonresponsive to empirical antifungal therapy.

Although the repertoire of the anti-*C. auris* properties of AMPs is yet to be fully explored, the foregoing clearly indicates their potential therapeutic utility. Although antimicrobial potential of AMPs has been explored for decades, their further development for clinical use appears to be notably slow as shown by the absence of any such, clinical trials at present. Therefore, further in-depth studies are warranted to explore, refine, and establish these AMPs as effective therapeutic agents against *C. auris*.

## Immunotherapy

Antibodies and various other immune molecules have been used over the last few decades to manage infectious diseases. Hence, some workers have attempted to use this approach for treating *C. auris* infections using antibodies and interleukins, with some degree of success.

### Anti-CR3-RP polyclonal antibody

It is well known that *Candida* species express a multitude of surface proteins to survive in hostile habitats and to enrich their biofilm lifestyle. Complement receptor 3-related protein (CR3-RP) is one such surface protein expressed by many *Candida* species including *C. auris*. Manipulation of the expression of these surface antigens have been shown to be effective in controlling *C. albicans* and *C. dubliniensis* biofilms.^105^ A study by Dekkerová *et al.* (2019) suggested that blockage CR3-RP expression through an anti-CR3-RP polyclonal antibody could disrupt *C. auris* biofilms development (36–73% inhibition) as well as established biofilms (28–46% inhibition) to a level comparable to those of fluconazole, amphotericin B, and caspofungin.^106^ These preliminary data implies that antibody therapies against *C. auris* infections may be feasible in the future.

### Antibodies against agglutinin-like sequence-3 (Als3p)

Using an approach similar to the above, Singh *et al.* (2019) demonstrated, in a mouse model, that antibodies against agglutinin-like sequence-3 (Als3 protein) surface adhesins of *Candida* species, i.e., anti-Als3p antibodies, could impede *C. auris* biofilm formation, and enhance the macrophage-mediated killing of the yeast.^107^ The anti-Als3p antibody formation in mice was triggered using a vaccine based on the N-terminus of Als3 protein. Importantly, the investigators observed that the vaccine was capable of inducing anti-Als3p antibodies (humoral immune response) as well as CD4 + T helper cells mediated activation of tissue macrophages (cellular immune response) that were highly effective against *C. auris* disseminated infections. In addition, co-administering the same vaccine in tandem with antifungal micafungin synergized the elimination of *C. auris* induced murine candidemia.^107^ Clearly, vaccines conferring lifelong immunity are the ultimate goal for protection against multidrug resistant yeasts including *C. auris*, and the foregoing brings this vision a step closer to realization.

### Interleukins

The armory of complex immune molecules that protect the body from invading pathogens, includes an array of various cytokines. Therefore, some have evaluated the antifungal properties of such agents against *C. auris*. In a preliminary study, Schneider *et al.* (2018) reported that human uterine cervical stem cells conditioned medium rich in several cytokines (IL-6, IL-8, IL-17, IP-10, CXCL-16, CCL-5, and CCL-6) with known antifungal activity could suppress fluconazole resistant *C. auris* isolated from a urine sample.^108^ Although it is not clear the specific components of the medium that elicited the inhibitory response, identifying such cytokines, may pave the way for developing immunological interventions against *C. auris* mucosal infections.

## Metals and nanoparticles

Elimination strategies of a new pathogen are primarily based on evaluating the effects of currently approved drugs, and repurposing those that are already approved against the other pathogens, against the newcomer. Essentially, this approach circumvents the extremely lengthy test protocols required for introducing a brand-new antimicrobial drug as a new therapeutic agent. Nevertheless, when all else fails, unconventional strategies of drug discovery have been increasingly experimented with a few recorded successes. This section addresses such approaches that have been taken for managing *C. auris* infections.

### Gallium

Certain metallic elements such as gallium have been proven to be valuable in managing cancers, and disorders of calcium and bone metabolism.^109^ In addition, compounds such as gallium nitrate (Ga(NO_3_)_3_) are known to be effective against various bacterial pathogens suggesting the possibility of their broader utility as antifungals.^110–113^ Their antimicrobial activity is due to the ability of gallium to replace iron, in iron containing proteins, thereby altering the functionality of the latter, arresting cellular metabolism and growth. In this context Gallium, acts through a ‘Trojan horse’ mechanism to alter the protein structure and the functionality of both bacterial and cancer cells.^113^

Bastos *et al.* (2019) were the first to investigate the effect of Ga(NO_3_)_3_ on *C. auris*, and they reported MICs ranging from 128 to 256 μg/ml for several strains of the multidrug resistant *C. auris* isolates. Although the MICs were considerably higher than *C. albicans* (16 μg/ml), the authors suggested its potential value as a fungistatic agent.^114^ However, the toxicity of such high gallium doses has not been evaluated thus questioning their value as a safe drug.

### Distorted gold(I)−phosphine complexes

Auranofin, a traditional anti-arthritis drug containing a gold complex, is known to possess anticancer, antibacterial, and antifungal properties.^115–117^ Dennis *et al.* (2020) studied six, distorted gold(I)−phosphine complexes against a panel of fungi including *C. auris* to evaluate their antifungal properties. Among the distorted compounds, chiral square-planar gold(I) complexes demonstrated strong anti-*C. auris* activity (MIC 0.98–7.8 μg/ml).^118^ The chiral square-planar gold(I) complexes were also fungicidal and exhibited excellent anti-biofilm activity (MBIC_90_ 3.9 μg/ml for developing biofilms, and 7.8–15.6 μg/ml against preformed biofilms). Unfortunately, the compounds were cytotoxic and elicited hemolysis indicating their limited potential, if any, in managing *C. auris* infections. Although the exact mechanism of their action is not yet known, it is likely that gold complexes may inhibit mitochondrial functions of the fungus.^119^

### Silver nanoparticles

Recent advances in nanotechnology and the realization that nanoparticles could be used as an effective drug delivery vehicle have led a number of workers to evaluate the potential of antimicrobial-laced nanoparticles in managing infectious diseases.^120^ Elemental silver, a key element that has been in use for many years as an antimicrobial agent due to its broad-spectrum antimicrobial activity has been used in this context, in the form of silver nanoparticles (AgNPs).

The latter is now known to possess potent activity against *C. albicans* biofilms.,^121,122^ Lara *et al.* (2019) observed a dose dependent effect of AgNPs (average size 1–3 nm) against *C. auris* and noted an IC_50_ of 0.06 μg/ml (0.06 ppm) for biofilm formation, and 0.48 μg/ml (0.48 ppm) for preformed biofilms. The biofilms treated with AgNP demonstrated cells with altered and, distorted morphology, and damaged cell walls.^123^ The investigators further demonstrated the potency of AgNPs in eliminating *C. auris* biofilm on colonized catheters, as well as on hospital fabrics. More importantly, the AgNP-interlaced fabric fibers (e.g., elastic bandage wraps) retained the fungicidal effect even after repeated washes indicating its lasting antifungal potency, and economy of use. The same group of workers, in a follow-up study, confirmed their previous findings (MIC < 0.5–1 μg/ml, MFC 1 ≤ 32 μg/ml), as well as the potency of AgNPs in preventing *C. auris* biofilm formation (IC_50_ 0.5–4.9 μg/ml) and eliminating preformed biofilms (IC_50_ 1.2–6.2 μg/ml) regardless of their clade. Interestingly, the biofilm phenotypes of all isolates were highly susceptible for AgNPs although a single *C. auris* isolate of one clade, showed a higher MFC value.^124^

It is believed that AgNPs exert their anti-candidal activity by attaching to the yeast cell surface, increasing the cell wall/membrane permeability, and disrupting the cell membrane integrity, leading to cellular apoptosis. In addition, others have noted a reduction of cell wall ergosterol content and hydrolytic enzyme production in *C. albicans* in response to AgNPs.^125–127^

### Bismuth nanoparticles

Similar to silver, elemental bismuth is also renowned for its antimicrobial properties including its anti-candidal properties, particularly against *C. albicans*.^128^ Vazquez-Munoz *et al.* (2020) demonstrated nanoparticles synthesized from bismuth (BiNPs), also exhibit promising anti- *C. auris* activity against planktonic *C. auris* regardless of their clades (MIC 1–4 μg/ml). However, its inhibitory effect on the biofilm phenotype appeared to be rather moderate as the IC_50_ values for biofilms ranging from 5.1 to 113.1 μg/ml.^129^

### Trimetallic nanoparticles

Nanoparticle comprising a combination of three components, so called trimetallic composites, such as silver copper and cobalt (Ag-Cu-Co), are now known to exhibit superior antimicrobial properties compared to their mono- and bi-metallic counterparts.^130^ The trimetallic nanoparticles also exhibit properties such as higher catalytic activity, better stability and selective and sensitive detection, increased drug encapsulation efficacy, in comparison to their monometallic equivalents.^131^ Kamli *et al.* (2021) recently investigated the effect of Ag-Cu-Co trimetallic nanoparticles against *C. auris* and noted a MIC range of 0.39–0.78 μg/ml against 25 different *C. auris* isolates. The authors also suggested that the nanoparticles are likely to exert their effect on *C. auris* by inducing cellular apoptosis and arresting its cell cycle.^131^

Thus, nanoparticle technology appears to show promise in combating *C. auris* both in infectious foci as well as in environmental ecosystems. Nevertheless, such applications will be premature until their PK/PD profiles, physicochemical interactions, toxicities, and their specific modes of action are determined.

### Silver nanoparticles and curcumin

Curcumin, well-known for its antimicrobial, anti-inflammatory, and antioxidant properties, has also been used in combination with AgNP as an antimicrobial agent.^132,133^ In particular, AgNPs laced with aqueous curcumin: hydroxypropyl-β-cyclodextrin complex (cAgNPs; average size 42.71 ± 17.97 nm), has shown to be effective against *C. auris*. Curiously though, free cAgNPs were toxic to mammalian cells, and poorly microbicidal, but when combined with a bacterial cellulose hydrogel, their cytotoxicity was reduced, and a significant antimicrobial effect was noted.^134^ Additionally, cAgNPs were found to be hemolytic, yet the Investigators suggested that loading such nanoparticles into bacterial cellulose hydrogel would be an attractive approach for eliminating pathogens such as *C. auris, Staphylococcus aureus* and *Pseudomonas aeruginosa* polymicrobial, chronic wound infections.

### Nanoparticle and nitric oxide (NO) combinations

Nitric oxide (NO), an important component of the mammalian innate immune system, possesses both cytostatic and cytotoxic properties against a broad-spectrum of microorganisms.^135,136^ The antifungal properties of NO have been previously demonstrated against *C. albicans*.^137^

In order to assess the anti-*C. auris* properties of NO, Cleare *et al.* (2020) developed nanoparticles that induce a sustained release of NO. They noted that 10 mg/ml of the nanoparticles were sufficient to completely arrest the growth of both fluconazole- susceptible and -resistant planktonic *C. auris* (a reduction of planktonic CFU by 1.49–10.2 log_10_). Interestingly, there was a significant reduction in biofilm viability when exposed to similar nanoparticle concentrations (a reduction of biofilm CFU by 0.98–9.68 log_10_).^138^ Of note, the nanoparticle scaffold itself displayed intrinsic inhibition of *C. auris* suggesting that the antifungal activity was a combined outcome due to the nanoparticle structure as well as the released NO.

Although NO nanoparticles are capable of disrupting fungal growth and morphogenesis by inducing cellular apoptosis and necrosis,^139^ there is no data on the cellular toxicity of the novel, combined formulations described above. Hence, further investigations are necessary to evaluate the potential clinical value of such combinations.

## Miscellaneous drugs and compounds

### Phenylthiazole family

Also, other molecules such as those from phenylthiazole family, with no previously reported antifungal activities, have been shown to eliminate *C. auris* planktonic phenotypes at 2 μg/ml (MIC) and display fungicidal activity, even after a short, 30-min exposure (2-4 folds of MIC).^140^ Another derivative of phenylthiazole family suppressed *C. auris* biofilm formation by 91.2% reduction at 2 μg/ml and reduced preformed biofilms by 50.7% at 8 μg/ml. Further, the phenylthiazole compound prolonged the survival of *C. auris* infected *C. elegans* nematode model and displayed no toxicity for mammalian cells indicating its potential as a future antifungal agent.^140^ Others have shown a related group of thiazoles, oxadiazolylthiazoles, and demonstrated anti- *C. auris* properties similar to phenylthiazole to (MIC 2-4 mg/ml).^141^

### MYC-053

Sodium 5-[1-(3,5-dichloro-2-hydroxyphenyl) methylideneam-ino]-6-methyl-1,2,3,4-tetrahydro-2,4-pyrimidinedionate, commonly known as MYC-053, appear to possess promising anti- *C. auris* properties against both fluconazole-sensitive and -resistant *C. auris* isolates. Although the exact mechanism of its activity is not known, it is suggested that MYC-053 may inhibit *C. auris* chitin synthase, that mediates cell wall chitin synthesis, and suppresses nucleic acid synthesis of the yeast and induces cell death.^142^

### VT-1598

VT-1598 is an investigational tetrazole with promising inhibitory properties against various fungi, particularly in experimental models of invasive candidiasis.^143–145^ Using *C. auris* isolates from South Asian, South American, East Asian, and African clades, Wiederhold *et al.* (2019) demonstrated that VT-1598 successfully eliminates the fungus both *in vitro* and *in vivo*. The overall MICs for all isolates ranged from 0.03–8 μg/ml (MIC50: 0.25 μg/ml and MIC90: 1 μg/ml). However, the isolates from South Asian clade displayed lesser sensitivity to VT-1598. When treated with up to 50 mg/kg, a longer survival rates (>21 days) and lower fungal burdens in the kidneys (mean log_10_ CFU/g, treated vs. control: 3.67 vs 7.26) were observed in a neutropenic murine model infected with *C. auris*.^146^

Despite the lack of *C. auris* specific data, VT-1598 is considered a selective inhibitor of fungal Cyp51A (Erg11p; 14α-demethylase) but not its mammalian counterpart of cytochrome *P*-450 enzymes.^147,148^ Thus, providing increased margin of safety for human use, if and when approved for such.

### Arylamidine T-2307

In a subsequent study, using similar experimental approach, the latter investigators demonstrated the anti-*C. auris* properties of another novel compound, arylamidine (T-2307). T-2307 destroyed 100% of planktonic *C. auris* at 0.125 to >4 μg/ml and significantly improved survival and reduced the kidney fungal burden in an animal model.^149^ T-2307 elicits mitochondrial membrane collapse in other fungi^150^ although, it is not yet known whether it deploys a similar mechanism for killing *C. auris.*

### Drimenol

In other published work, drimenol, a synthetic drimane sesquiterpenoids, was demonstrated to completely inhibit *C. auris* growth at 50 μg/ml (MIC 30 μg/ml).^151^ Although exact mechanism of *C. auris* inhibition is yet to be known, drimenol is likely to affect fungal cellular activities that regulate protein secretion, vacuolar functions, chromatin remodeling and cyclin dependent protein kinase (CDK)-associated functions.^151^

### 2-Aryloxazoline derivatives

Similarly, two of the 2-aryloxazoline derivatives generated from a reaction between L-threonine, and derivatives of naphthoic or salicylic acid also exhibited MIC of 0.06-2 μg/ml against fluconazole and amphotericin B sensitive and resistant *C. auris* isolates, respectively.^152^ However, their mode of action, tolerability/toxicity and in-depth PK/PD is yet to be determined.

### Cuminaldehyde derivative

Soliman *et al.* (2018) has shown a promising ant-*C. auris* activity of derivatives of cuminaldehyde, an essential oil isolated from *Calligonum comosum.* Cuminaldehyde is known for its antifungal properties, however, its toxicity has impeded its use as an antifungal agent.^153^ In order to improve its biocompatibility, Hamdy *et al.* (2020) synthesized cuminaldehyde derivatives by replacing the toxic aldehyde group of the essential oil. The derivatives elicited antifungal activity against *C. auris* at a concentration range of MIC_50_ 2-15 μg/ml, and the compounds were well tolerated by mammalian cells.^154^

### Amidinourease compounds

In another study, Orofino *et al.* (2020) noted the putative anti- *C. auris* properties of a synthetic compound from macrocyclic amidinourease, a novel class of antifungal agents. They exhibited activity against fluconazole resistant strains of *C. auris*, with a MIC range of 8–64 μg/ml, and were well tolerated in a murine model. The exact mechanism of their action is yet to be determined.^155^

### Aryl- and heteroaryl-substituted hydrazones

Thamban-Chandrika *et al.* (2021) recently proposed a potential new class of antifungal agents, fluorinated, aryl- and heteroaryl-substituted hydrazones.^156^ When an array of synthetic monohydrazones (family IV) were screened for their antifungal properties against *C. albicans* and a panel of *C. auris* isolates (n = 10), they noted seven compounds to be highly active against all *C. auris* isolates with a MIC 0.015–7.8 μg/ml. Two of the novel compounds significantly suppressed biofilm formation (15.6–31.3 μg/ml; 4-16x MIC) as well and exhibited better MBIC_50_ than voriconazole. These compounds appeared to be well tolerated by the host cells as murine red blood cells were not adversely affected when exposed to these monohydrazones.^156^ Although the mechanisms of action of the monohydrazones are yet to be unraveled, the latter authors suggested that they are likely to interfere with fungal DNA-protein interactions.

### Acetohydroxyacid synthase inhibitors

Acetohydroxyacid synthase (AHAS) has been used as the target for over 50 commercial herbicides and is considered a promising target for antimicrobial drug discovery. Garcia *et al*. (2018) suggested the suppression of AHAS, the first enzyme in the branched-chain amino acid biosynthesis pathway, as an effective strategy for managing invasive antifungal infections.^157^

Using purified AHAS derived from *C. auris*, Agnew-Francis *et al.* (2020) identified 13 different AHAS inhibitors. Among these, bensulfuron methyl appeared to be the most potent (MIC_50_ of 0.09 μM) and fungicidal against two *C. auris* strains tested. In addition, both bensulfuron methyl and chlorimuron ethyl, exhibited potent antibiofilm effects (MBIC_50_ 0.596–1.98 μM).^158^ Several AHAS inhibitors identified in this study (chlorimuron ethyl, bensulfuron methyl, metosulam, diclosulam, cloransulam methyl) maintained growth inhibition for a period of 14 days indicating their potential long-term efficacy.^158^ The AHAS inhibitors have exceptionally low toxicity against mammalian cells,^159^ and could prove to be an another viable therapy for managing *C. auris* infections.

The foregoing clearly indicates the explosion of interest in the drug discovery against *C. auris*, and the multitude of agents evaluated as anti-*C. auris* agents, although, unfortunately, none appear to have been approved for therapeutic use, thus far. Further, in depth*, in vitro* and *in vivo* studies, particularly with broader coverage of all its clades form different geographic regions, are essential to achieve this ultimate goal.

## Natural compounds

Apart from the synthetic compounds described above a number of natural metabolites derived from microbes have been another focus of study in the quest for anti-*C. auris* agents, and these are described below.

### Quorum sensing molecules (QSMs) – Farnesol

Microbial quorum sensing (QS) is the phenomenon of gene expression within a microbial community through chemical signals, termed QS molecules, in response to fluctuations in communal cell-population density.^160^ QSMs are also known to possess potent antimicrobial properties, particularly against competing organisms within microbial communities, and this attribute has been exploited in the search for potential antimicrobial agents.^161–163^

Farnesol, for instance, a major QSM secreted by some *Candida spp.* inhibits the morphological transition of *C. albicans* and non-*albicans Candida* species from the yeast to the hyphal phase thereby impeding their virulence.^164–166^ Interesting in the current context is the work of Nagy *et al.* (2020) who demonstrated concentration dependent (from 10 to 300 μM) inhibition of *C. auris* biofilm growth and development, up to 24 h.^167^ The latter researchers also reported the synergism between farnesol and triazole antifungals (fluconazole, voriconazole, isavuconazole, itraconazole and Posaconazole), on preformed 24 h *C. auris* biofilms, (with a FICI ranging from 0.038 to 0.375). In an earlier study, the same group reported synergism between farnesol and three echinocandins; anidulafungin, caspofungin, or micafungin in inhibiting biofilms of four *C. auris* isolates of South Asian/Indian lineage.^168^ They witnessed 64-128 folds reduction in biofilm inhibitory concentrations (≥64 μg/ml vs. 1 μg/ml) of all three echinocandins (by means of 50% reduction in biofilm metabolism) when treated with farnesol.^168^

Using 25 different *C. auris* isolates from South Africa, including 22 fluconazole and five amphotericin B resistant strains, Srivastava *et al.* (2020) demonstrated similar inhibitory properties of farnesol on planktonic and biofilm phenotypes of the yeast, although the overall inhibitory concentration of the QSM was markedly higher than noted in previous studies.^167–169^ The researchers observed planktonic MIC of farnesol ranged between 62.5 and 125 mM. Farnesol concentrations of 125 mM inhibited *C. auris* adhesion, 7.81 mM inhibited >50% of forming biofilms, and 500 mM inhibited 12 and 24 h biofilms.^169^ As regards the morphologic effects of farnesol on *C. auris* biofilms, Srivastava *et al.* (2020) noted thin, scanty, and sparse biofilm development on exposure to farnesol, in comparison to thicker and robust biofilm growth in farnesol-free controls.^167,169^

The exact mechanisms by which farnesol impacts *C. auris* planktonic and biofilm phenotypes are not yet known, although, it has been speculated that the reduced activity of drug efflux pumps and the downregulation of the genes coding them (*CDR1, CDR2* and *SNQ2*) may be the reasons for such observations.^169^ It is well established that farnesol is actively involved in ergosterol biosynthesis, induce intracellular ROS, thus disrupting mitochondrial metabolism in several *Candida* species.^170^ Hence it is highly likely that the inhibitory properties of QSM against *C. auris*, including the synergism with echinocandins, are likely to be associated with one or more of these mechanisms. Further work is warranted however, to confirm the foregoing hypotheses, evaluate the bio- safety, and realize the promising potential of farnesol as an anti *C. auris* agent.

### Chitosan

Chitosan (poly-(β-1→4)-2-amino-2-deoxy-D-glucopyranose), a naturally occurring, nontoxic polymer derived from deacetylated chitin, commonly found in fungal cell walls, as well as in crustacean exoskeletons is known to exhibit broad spectrum antimicrobial activity.^171^

Chitosan has been tested against both aggregating and non-aggregating phenotypes of *C. auris* (from Southern Asian/Indian and South African clades) for their anti- candidal properties.^172^ The latter study reported that fungicidal concentrations of chitosan for planktonic *C. auris* ranged between 5–20 μg/ml, while for the biofilm phenotype MIC50 and MIC80 ranged between 10 and 80 μg/ml and 40 and 160 μg/ml, respectively. Two aggregating phenotypes with known resistance to caspofungin exhibited the highest planktonic and sessile MIC.^23^

In ultrastructural studies, the chitosan treated non-aggregating phenotype, unlike the aggregating counterpart, exhibit ruptured cell walls, implying phenotype dependent response of *C. auris* to the polymer.^172^ Further analyses of the differential expression of genes associated with stress-like response (*ALS5, HYR3, ERG2, KRE6, EXG, ENG1, SAP5, PLB1*), the latter researchers observed upregulation of all genes in non-aggregating phenotype (only *SAP5* in aggregating phenotype). In another study, chitosan treatment (200 mg of chitosan/kg of body weight) significantly increased the survival rate (up to 84%) of *G. mellonella* wax worm infected with *C. auris* compared to untreated controls. Although the link between the chitosan exposure and the foregoing gene expression is unclear, particularly for aggregating and non-aggregating phenotypes, the investigators suggested that the direct interactions of chitosan with the cell surface of the fungus may have contributed to this phenomenon.^172^ Nevertheless, *C. auris* inhibitory properties of chitosan appear promising and further investigations are warranted to confirm their clinical utility as a therapeutic agent.

### Plant products

Medicinal plants, plant products and essential oil derivatives have been used for millennia as components of various traditional medicines, due to their potent antimicrobial activities.^173–176^ The active anti-microbial ingredients of these plant products are complex and varies intensely but are minimally toxic to human cells. In general, the active components of the herbal products are monomers such as phenylpropanoids, flavonoids, alkaloids, terpenoids, and quinones.^177,178^ The following section summarizes some of the plant products evaluated as anti- *C. auris* agents.

#### Herbal monomers

Liu *et al.* (2020) explored the anti- *C. auris* properties of five common traditional herbal monomers used in Chinese medicine, berberine, sodium houttuyfonate, jatrorrhizine, palmatine, and cinnamaldehyde, that are already known to be antifungal in nature.^179^ They reported planktonic MICs of 64 μg/ml for sodium houttuyfonate, and 50 μg/ml for cinnamaldehyde, in contrast to the other three monomers which displayed higher MIC values of 256 μg/ml. The investigators also noted a significant reduction in *C. auris* colonization and aggregation, and a greater degree of cell wall β-glucan exposure in response to a combination of cinnamaldehyde, jatrorrhizine, and palmatine, as opposed to exposure to the individual compounds. Although the exact mechanism is not known, their effects are thought to be associated with either the cell wall development mechanics and/or the fungal stress response. This hypothesis is further supported by the cell wall remodeling properties of some of the traditional herbal monomers.^180^

#### Cinnamaldehyde

The essential oil extracted from *Cinnamomum zeylanicum* Blume (Sri Lankan cinnamon) leaves and bark have been widely studied for their antiseptic, immunostimulant, detoxifying, analgesic, and antidepressant effects. Previous studies have shown the potential of this essential oil in inhibiting germ tube formation, adhesion to epithelial cells and proteinase production in *C. albicans*.^181^ Essential oil extracted from cinnamon bark that contains (∼66%) *trans*-cinnamaldehyde is now known to be fungicidal at 0.03% (v/v) concentrations, and to lower *C. auris* associated hemolysis.^182^ The latter workers further suggested that the active compound, cinnamaldehyde is likely to exert its activity by compromising the yeast cell membrane and cell wall integrity.

#### α-Cyperone

An essential oil containing α-Cyperone extracted from rhizome of *Cyperus rotundus* seem to possess beneficial properties such as protecting host cells from lipopolysaccharide mediated cellular damage and from H_2_O_2_-induced oxidative stress and apoptosis in neuronal cells.^183^ Recent study by Horn *et al.* (2021) demonstrated the inhibitory properties of α-Cyperone on *C. auris* growth at a concentration of 150 μg/ml.^184^ Their anti-fungal mechanism is yet to be elucidated.

#### 6-Shogaol

6-shogaol is a biologically active phytochemicals in ginger and displays potent *C. auris* anti-biofilm activity. 6- shogaol has a low MIC (MIC_50_ 16–32 μg/ml, and MIC_80_ 32–64 μg/ml) for the planktonic form, with over 97% suppression of forming and preformed biofilms of *C. auris* on exposure to 64 μg/ml. Although the molecular mechanism of its anti- *C. auris* activity is yet to be defined, the authors suggested that 6- shogaol is likely to act on drug efflux machinery of the fungus.^185^

Clearly, not all the tested essential oils or their active compounds seem to possess effective and strong anti- *C. auris* properties. For example, an essential oil extracted from native eastern north American plant *Thuja plicata*, commonly known as American arborvitae, exhibited only a marginal reduction in intrinsic growth rate of *C. auris*, possibly due to increased cellular death or decreased cell division.^186^

From the foregoing it is evident that plant oils, extracts and monomers have significant, yet highly variable anti*- C auris* properties. Further in-depth investigations into such phytochemicals with promising potential should pay dividends in the quest for natural and green, pharmacological products that are antifungal in nature.

### Bee honey

Medical-grade honey contains various phytochemicals such as alkaloids and flavonoids, in addition to bee-derived peptides, such as bee-defensin-1 and apidaecin.^187,188^ Honey is also known to have over 200 different components that may vary from one shipment to another depending on the source of origin, the geographical location, as well as the bee species.^187^

Most of the honey constituents are known to possess antimicrobial properties although these may vary among different formulations. Despite the latter drawback, de Groot *et al.* (2021) tested a medical grade honey formulation against 32 isolates of *C. auris* from five different clades and observed that exposure to 40% honey for 24 h results in a notable reduction in CFU counts (by 2 Log_10_). However, as pure honey, at similar concentrations, had inferior activity, the authors concluded that the observed yeast inhibition was likely to be due to a combined effect of honey components, as well as other commercial additives in their samples.^189^ Although bees honey may be a promising anti-*C. auris* candidate compound, quality control of such naturally derived products would be a major impediment that needs to be overcome in future studies.

### Probiotics

Probiotics therapy is now a widely accepted alternative strategy for improving the overall human health due to their beneficial, synergistic interactions with the commensal flora while inhibiting colonisation by extraneous pathogens. In this context, probiotics have been studied for their potential in preventing and managing fungal infections including those caused by *Candida spp*.^190,191^

Rossoni *et al.* (2020) investigated the impact of the probiotic *Lactobacillus paracasei* 28.4 and its supernatant on *C. auris.* The co-culture of the probiotic with *C. auris* led to a significant reduction of yeast counts for up to 3 days. The crude extracts of the supernatant (>15 mg/ml) and its first fraction (3.75 –>7.5 mg/ml) also demonstrated a significant suppression of all 10 *C. auris* isolates tested (up to 6 log_10_ CFU reduction).^192^ In a subsequent study, co-inoculation of *C. auris* with two different probiotic yeasts; *Saccharomyces cerevisiae* and *Issatchenkia occidentalis* resulted in reduction of *C. auris* adhesion by 44–62%.^193^ Additionally, both the probiotic and the supernatant were effective in impeding *C. auris* biofilm growth, significantly prolonged the survival of *C. auris* infected *G. mellonella*.^192^

As the anti-fungal components of the probiotic supernatants are yet to be deciphered, futures workers should seek to isolate, identify, and characterize these bioactive molecules to evaluate their translational potential as therapeutics against *C. auris.* Probiotics are particularly attractive potential therapeutics as they are generally regarded as safe by FDA and have been tested for their clinical efficacy against *C. albicans*.^194^

## Novel compounds with antifungal properties

### ‘*Fungerp’* group of antifungal agents

Since the first reports of the stubborn resistance profile *C. auris* to the current antifungal armamentarium, a number of workers have attempted to evaluate novel anti- *C. auris* compounds, and as a result, significant new discoveries have been made, as discussed below.

Several studies have been conducted for evaluating compounds that are structurally and/or functionally similar to the most effective family of anti-*C. auris* drugs, namely glucan synthase inhibitors such as echinocandins. One such compound, so called ‘*fungerp’* antifungal agents, has been tested against *C. auris*, is ibrexafungerp (*syn*. SCY-078) is an orally bioavailable triterpene glucan synthase inhibitor. It is known to possess antifungal properties against common pathogenic *Candida* species, including those resistant to echinocandins.^195^

Berkow *et al.* (2017) demonstrated the efficacy of SCY-078 against a panel of 100 different *C. auris* isolates from four known clades from India, Pakistan, Colombia, South Africa, and the United States.^15,196^ The MIC values of SCY-078 in these studies ranged from 0.0625 to 2 μg/ml (mode MIC_50_ 0.5 μg/ml and MIC_90_ 1 μg/ml).^196^ Importantly, seven isolates that were resistant to anidulafungin, caspofungin or micafungin exhibited susceptibility to SCY-078 (MIC 0.5–1 μg/ml). Zhu *et al.* (2020) have also confirmed these MIC profiles in a large-scale screening study of 200 different *C. auris* isolates.^197^ Using 16 isolates, Larkin *et al.* (2017) further demonstrated the potent antibiofilm activity of the new compound is likely to be due to the reduction of the metabolic activity as well as the thickness of *C. auris* biofilms treated with SCY-078. These investigators also suggested that the compound is likely to act on different cellular targets as they observed, in SEM studies, severely altered yeast morphology and cell division arrest in SCY-078 treated *C. auris*.^198^

In addition to being bioavailable orally, SCY-078 differs from other echinocandins as its activity is not compromised by the most common yeast mutations within the protein target, Fks.^199^ This has been confirmed in a study conducted on a panel of 122 *C. auris* isolates from various geographical clades. There were eight echinocandin resistant isolates within the tested panel of the isolates with a S639F Fks1 alteration. Interestingly, all 122 isolates were susceptible to SCY-078 with a modal MIC and MIC_50_ of 0.5 μg/ml (a range of 0.06–2 μg/ml).^200^ In practical terms, the range of MICs reported in these studies is well within the serum concentrations recorded in murine models of disseminated candidiasis, and preclinical pharmacokinetic and pharmacodynamic (PK/PD) studies.^201^

Ghannoum *et al*. (2020) demonstrated using a guinea pig model, that oral administration of (10 mg/kg) of SCY-078 lowers the severity of lesions as well as the fungal burden in infected animals compared to the controls.^202^ Therefore, SCY-078 is highly likely to be one of the more promising new antifungal agents against echinocandin-resistant *C. auris* infections, and currently it is undergoing phase III clinical trials (NCT04029116).^203^

The potent antifungal activity of SCY-078 has led to further experiments using its analogs SCY-247. One such second-generation ‘*fungerp’* antifungal compound, elicited an MIC range of 0.06–1 μg/ml; MIC_50_ and MIC_90_ 0.5 μg/ml which was comparable to SCY-078, against a panel of different *C. auris* isolates.^204^ Interestingly, SCY-247 exhibited fungicidal effects (MFC range 0.5–8 μg/ml; MFC_50_ and MFC_90_ 4 μg/ml) on a larger percentage of *C. auris* isolates than SCY-078 (14 vs. 7 isolates). Further investigations on SCY-247 *in vivo* responses, the potential for developing resistance, the molecular mechanisms of action, and their side effects will be important to validate the fitness of SCY-247 as an anti-*C. auris* antifungal agent. In summary, the family of fungerp antifungal compounds SCY-078 and its analogs are likely to be a potent antifungal drug class in future.

### Fosmanogepix or APX001/APX001A

APX001/APX011A (Fosmanogepix, formerly E1211), a water-soluble small molecule with a novel and unique mechanism of action, has drawn the attention of a number of workers searching for effective therapies against *C. auris*. Unlike other classes of antifungal agents, the active moiety of the APX001, which is released by its rapid metabolism by systemic phosphatases, targets a highly conserved fungal enzyme Gwt1 (glycosylphosphatidylinositol-anchored wall transfer protein 1). Gwt1 catalyses the inositol acylation step of glycosylphosphatidylinositol (GPI) anchored cell wall mannoproteins synthesis. These mannoproteins play a significant role in anchoring the fungus to eukaryotic cell surface proteins.^205,206^ The inhibition of Gwt1 is known to affect maturation and localization of fungal cell wall mannoproteins, leading to compromised cell wall integrity, defective filamentation and biofilm formation, and severe retardation of fungal growth.^205,207^ Interestingly, the PIG-W protein, the mammalian ortholog of Gwt1, appeared to be insensitive APX001 mediated inhibition, thus, significantly improving the fungal specificity of the drug target.^205^

One of the early studies conducted by Hager *et al.* (2018) on the effects of APX001 on 16 isolates of *C. auris* have shown that all isolates were susceptible to the novel compound, with MIC_50_ and MIC_90_ values of 0.004 and 0.031 μg/ml, respectively.^208^ In another study, using a large array of *C. auris* isolates (n = 100) from four different geographical clades, Berkow and Lockhart (2018) further confirmed and validated these results across the clades (MIC range <0.005–0.015 μg/ml, overall modal MIC 0.005 μg/ml, MIC_50_ 0.002 μg/ml and MIC_90_ 0.008 μg/ml).^209^ The efficacy of APX001 against six echinocandin resistant isolates included in this study suggests its value in treating echinocandin resistant *C. auris* infections. Furthermore, Arendrup *et al*. (2018) also noted in an *in vitro* study that APX001 is highly effective against a collection of 122 *C. auris* isolates (MIC_50_ = 0.016 μg/ml).^210^ Their data also indicated that APX001 was equally or more active than anidulafungin, micafungin, voriconazole, fluconazole, and amphotericin B.^197,200,210^

APX001 has been proven to be very effective in *in vivo* models. For instance, the exposure of immunocompromised mice infected with *C. auris* to APX001 resulted in a significantly higher 16-day survival rate (as high as 100%) compared to the treatment with anidulafungin. APX001 treated mice had significantly lower CFU counts in kidney, lung, and brain tissue (with a log_10_ reduction range of 1.03–1.83) versus the vehicle control.^208^ In a parallel study, using a similar disseminated candidiasis murine model, Zhao *et al.* (2018) confirmed the efficacy of the new compound in eliminating *C. auris* infection *in vivo* and described that the outcome was dependent on the concentration of APX001.^211^ The foregoing clearly testifies to the fact that APX001 is likely to be a highly effective compound against *C. auris* infections. Indeed, as a novel drug, currently in the clinical development phase, (https://clinicaltrials.gov/ct2/show/NCT04240886), APX001/APX001A would likely to be one of the key last resort drugs in managing multi-resistant *C. auris* infections in the not-too-distant future.

### New echinocandin agents – Rezafungin (CD101)

Rezafungin (CD101) is yet another echinocandin currently in the clinical development pipeline. Compared to current echinocandins, rezafungin offers enhanced pharmacokinetic properties and has an improved safety profile,^212–214^ and the efficacy of this compound is proven against isolates of several other *Candida* species.^215–217^

In one such study, Berkhow and Lockhart (2018) noted that MIC values of rezafungin ranged between 0.03 and 8 μg/ml (mode MIC50 = 0.125 μg/ml, MIC90 = 0.5 μg/ml) among 100 different *C. auris* isolates. Similar to previous investigations with SCY-078 and APX001A, there were no notable variations among four clades tested. However, 4 of 8 echinocandin resistant isolates exhibited higher MICs to rezafungin (MIC range 0.06–8 μg/ml with an MIC_50_ of 0.5 μg/ml).^218^ Further analyses revealed this to be due to S639P amino acid substitution in Fks1 hot spot 1, a mutation corresponding to the echinocandin resistance in other *Candida* species (e.g., S645P in *C. albicans* and S629P in *C. glabrata*).^219^ Generally, the relative increase in the MIC of rezafungin conferred by Fks mutations was comparable to or slightly less than those for anidulafungin and micafungin.^218,220^ In accordance with cross resistance between rezafungin and the comparator echinocandins observed previously, Helleberg *et al.* (2020) also reported increased MIC of rezafungin in *C. auris* isolates with Fks1 hot spot S639F mutations (MIC 8–16 μg/ml).^220^ However, this increase in MIC was 3–4-folds greater than for the anidulafungin and micafungin. It is clear that FKS mutations would likely to impact the efficacy of rezafungin and the increase in the rezafungin MIC is likely to be dependent on the codon and the substitution involved in the mutation. Therefore, further investigations are warranted in understanding rezafungin resistance in *C. auris.* On a positive note, the azole resistance in *C. auris* did not appear to impact the efficacy of rezafungin.^221^

Rezafungin has also displayed some encouraging results in *in vivo* disseminated *C. auris* candidiasis models. Using a murine model, Hager *et al.* (2018) demonstrated potent reduction of *C. auris* in kidney tissues compared with controls, and those treated with amphotericin B, up to 10 days posttreatment and compared to those treated with micafungin on 10th day posttreatment.^222^ In a similar disseminated candidiasis murine model, Lepak *et al.* (2018) further validated the efficacy of rezafungin in eliminating *C. auris* infection *in vivo*. By integrating their pharmacokinetics/pharmacodynamics (PK/PD) targets with human PK studies, the investigators further suggested that intravenously administered rezafungin dose of 400 mg once a week would likely to meet or exceed the PD target for >90% of *C. auris* isolates.^223^

To conclude, rezafungin shows promising activity against *C. auris* and its once-weekly intravenous therapy would likely to be an attractive therapeutic strategy for critically ill with the fungal infection. The prolong half-life, greater safety margin with chemical stability provide rezafungin a notable advantage of preventing the development of resistance to echinocandins class of antifungal agents. Large scale clinical investigations are, however, needed to translate these findings into the therapeutic domain.

### Novel azole antifungal agents –PC9454

Due to the very high safety margin and good biocompatibility, the efficacy of novel azole antifungals against *C. auris* has also been evaluated.^26^ The efficacy of one such novel triazole antifungal, 4-[4-(4-{[(3R,5R)-5-(2,4-difluorophenyl)-5-(1H-1,2,4triazol-1-ylmethyl)oxolan-3-yl]methoxy}-3-methylphenyl) piperazin-1-yl]-N-(4-fluorophenyl) benzamide, also known as PC945, was tested against 72 *C. auris* isolates from India, UK, Japan, S. Korea, and USA.^224^ They noted that overall, respective MIC_50_ and MIC_90_ of PC945 to be 0.063 and 0.25 μg/ml against *C. auris* suggesting its superior potency. In fact, PC945 was 7.4- and 1.5-fold more potent than voriconazole and posaconazole, respectively.

PC945, similar to other azoles, acts on ergosterol synthesis pathway by inhibiting lanosterol 14a-demethylase enzyme coded by *ERG11*. Yet the data indicated that PC945 acts independent of any mutations in the *ERG11* that may be associated with azole resistance in *C. auris*, indicating its superior effectiveness over other azoles against azole resistant *C. auris*.^224^

### Novel candidate agents - Ebselen

Rather than following the conventional route of repurposing the currently available antimicrobials, some have taken the more challenging route of evaluating totally new compounds for their antifungal activity. In one such study, by screening a library of 1 280 small molecules, Wall *et al.* (2018) identified nine candidate molecules with potential antifungal activities against *C. auris*.^225^ One of these molecules with no recorded antifungal activity, to date, Ebselen (2-phenyl-1,2-benzisoselenazol-3(2H)-one), exhibited 100% growth inhibition of *C. auris* at physiologically achievable concentrations (as low as 2.5 μM).^225–227^ These investigators further noted that ebselen actively impedes the biofilm phenotype of the fungus at physiological concentrations of 5.9 to 9.8 μg/ml. The novel molecule appeared to be highly effective against *C. auris* irrespective of its resistance to fluconazole, amphotericin B or caspofungin. Although its exact antifungal mechanism is not yet known, ebselen is known to induce ROS mediated cytotoxicity and the membrane H^+^-ATPase pump (Pma1p) in *Saccharomyces cerevisiae*, and also deplete the fungal intracellular glutathione levels.^228,229^ Same investigators have also noted synergistic interactions between ebselen and anidulafungin.^225,230^

### Novel candidate agents - Miltefosine and Iodoquinol

Wall *et al.* (2019) in a subsequent study, identified two further compounds, miltefosine and iodoquinol, with anti- *C. auris* properties by screening another chemical library (Pathogen Box®). They noted the potent *in vitro* inhibitory activity of iodoquinol against planktonic *C. auris*, and miltefosine against both planktonic and biofilm phase *C. auris* at 4 μg/ml, irrespective of the antifungal resistance profiles of the chosen isolates.^231^ The same group of researchers in another study, identified 26 different compounds with anti- *C. auris* activity by screening of over 12 000 small molecules (ReFRAME library). These included antiseptics, disinfectants, antibacterials and most interestingly five repositionable compounds including miltefosine (Tazomeline, Lonafarnib, AM-24, Miltefosine and Provecta). However, the mechanisms of antifungal actions of the latter compounds are yet to be defined.^232^

### Novel candidate agents - Suloctidil

Concurrently, de Oliveira *et al.* (2019), by screening another chemical library (Prestwick Chemical Library), identified 12 different compounds with ≥90% growth inhibition of *C. auris*, and seven of these demonstrated reproducible antifungal activities. These were suloctidil, trifluoperazine dihydrochloride, ciclopirox ethanolamine, tamoxifen citrate, ebselen, pyrvinium pamoate, and thiethylperazine dimalate.^230^ Among these, suloctidil inhibited *C. auris* growth by >78% at a concentration of 16 μg/ml. They also demonstrated a synergy between suloctidil and voriconazole, that led to 2-32-fold lowering of MIC of voriconazole against *C. auris*.^230^

### Novel candidate agents – Niclosamide and Halogenated Salicylanilide

Furthermore, a screen of a chemical library of 678 small molecules revealed that niclosamide (5-chloro-salicyl-(2-chloro-4-nitro) anilide), an FDA-approved anthelmintic drug for humans, and a halogenated salicylanilide (N1-(3,5-dichlorophenyl)-5-chloro-2-hydroxybenzamide), another anthelmintic drug for veterinary use, as compounds with potential antibiofilm properties against *C. auris.* Both anthelmintic drugs demonstrated anti- *C. auris* biofilm properties at 1 μM. Although their *C. auris* specific molecular mechanisms are yet to be known, the two anthelmintic drugs are known to subdue *C. albicans* virulence suppressing morphological transition and mitochondrial protein import machinery.^233^

## Repurposed drugs

As discussed above, screening compound libraries have revealed hitherto unknown non-antimicrobial drugs such as miltefosine, niclosamide, and halogenated salicylanilide with promising antimicrobial potential.^232,234^ As a consequence, there has been a renewed interest in repurposing older drugs as antifungal agents. For instance, Hao *et al.* (2020) witnessed the potent inhibition of *C. auris* planktonic phase by 4–8 μg/ml of disulfiram, a drug used for treating chronic alcoholism, however, its ability to inhibit the biofilm phenotype appeared to be modest (MBIC_80_ 64–128 μg/ml).^234^ The following section discusses some key non-antimicrobial drugs that could be repurposed and exhibited promising anti-*C. auris* properties.

### Sertraline

In another work, Gowri *et al.* (2020) reported that an antidepressant sertraline is capable of suppressing *C. auris*.^235^ The MIC of sertraline against three different *C. auris* isolates ranged between 20 and 40 μg/ml. The antidepressant exhibited its fungicidal activity as early as 6 h, and suppressed biofilm formation by 71%, at doses of 20 μg/ml. Through *in silico* studies, authors noted that sertraline elicits its deleterious effect on *C. auris* by binding to the Erg11p in the ergosterol biosynthesis pathway, as they noted a 32-fold reduction in the ergosterol content of the test samples.^235^ The anti- *Candida* properties of sertraline have been previously recorded, and findings of this study further validates its broad spectrum of activity although the collateral repercussions of administering such doses to those that are otherwise healthy needs further investigations.^236^

### Alexidine dihydrochloride

Alexidine dihydrochloride (a bis-biguanide dihydrochloride), an anticancer drug that targets a mitochondrial tyrosine phosphatase in mammalian cells which drive mitochondrial apoptosis,^237^ has been noted to inhibit *C. auris* by Mamouei *et al.* (2018). They reported >80% inhibition of fluconazole resistant planktonic phase (at 1.5 μg/ml), and developing and mature biofilm phase (at 3–6 μg/ml) of *C. auris* when exposed to alexidine dihydrochloride.^238^

The anticancer drug appeared to be well tolerated by mammalian epithelial cells (5–10× planktonic MIC is needed for 50% killing of HUVEC), although its toxicity for some immune cell components such as macrophages, appeared to be high (50% cytotoxic concentration, CC_50_, of over 5 μg/ml).^238^ Interestingly, the drug is already used in dentistry as an antiplaque agent and root canal irrigant due to its antibacterial properties.^239–241^ Therefore, its further optimization into a compound with a higher efficacy and bioavailability, and low toxicity would likely to generate a successful repurposed antifungal agent.

### Mefloquine derivatives

In another study that investigated the potential of repurposed approved drugs, derivatives of mefloquine, an orally prescribed, 4-quinoline-methanol antimalarial drug, was noted to possess anti- *C. auris* properties. Montoya *et al.* (2020) tested a small group of mefloquine derivatives against fluconazole susceptible and resistant *C. auris* and noted their planktonic MICs to range between 2 and 8 μg/ml.^242^ The derivatives were also effective against fluconazole resistant *C. auris* isolates (MIC 4-8 μg/ml). Interestingly, despite this observation mefloquine itself was largely ineffective against a tested *C. auris* isolate (MIC 128 μg/ml). They suggested that the antifungal effect of the derivatives may be associated with their ability to disrupt the mitochondrial membrane, vacuolar disruption and interfere with DNA stability – a mode of action distinctive from existing antifungal drugs.^242^ As the antifungal activity of these derivatives have been previously shown against with *Cryptococcus neoformans* and *C. albicans*^243^ further optimization of their pharmacokinetics is likely to be fruitful.

## Perspectives and concluding remarks

The multitude of reports on the new therapeutic strategies against *C. auris* infections reported here is rather startling, considering the fact that the organism was first described over a decade ago in 2009. Its alarming global spread, multi- resistance to almost all of the currently available antifungals, and the morbidity and mortality it causes are the clear reasons for such great interest in this inveterate `new kid on the block'. Fortunately, there is hope, as a vast majority of compounds reported thus far appear to exhibit encouraging anti- *C. auris* properties, with promising drugs now in the pipeline in various stages of development. Nevertheless, there are several areas that need immediate attention in anti- *C. auris* therapeutics. The lack of data on the modes of action, toxicity, dosage, and the potential of *C. auris* to develop resistance to the new therapeutic modes is clear, and further studies are urgently warranted. As per EU Clinical Trials Register and NIH ClinicalTrials.gov, several novel antifungal compounds are currently undergoing clinical trials, however, only two of the aforementioned compounds (SCY-078, APX001) have been specifically tested on *C. auris*. Considering the severity of the infection and the incidence of antifungal resistance of C. auris, urgent attention is needed to minimize the lag between laboratory testing and clinical validation of anti-*C. auris* compounds. This yeast is also unique in its bi-pronged action of being a harmful human pathogen, and unlike most of its counterparts, possessing the ability to survive in the environment, and fomite surfaces, for weeks, maintaining its ability to cause infection. Hence strategies to tackle the organism should include not only the development of appropriate antifungal therapeutics, but also good environmental disinfectants effective in healthcare ecosystems. Finally, due to the isolation of the pathogen in over 45 different countries, future studies are needed with an inclusion of wide range of clinical isolates and a greater clade representation so as to produce data of grater relevance, significance, and validity.
